# A Proton Magnetic Resonance Spectroscopy (^1^H MRS) Pilot Study Revealing Altered Glutamatergic and Gamma-Aminobutyric Acid (GABA)ergic Neurotransmission in Social Anxiety Disorder (SAD)

**DOI:** 10.3390/ijms26146915

**Published:** 2025-07-18

**Authors:** Sonja Elsaid, Ruoyu Wang, Stefan Kloiber, Kimberly L. Desmond, Bernard Le Foll

**Affiliations:** 1 Translational Addiction Research Laboratory, Campbell Family Mental Health Research Institute, Centre for Addiction and Mental Health, Toronto, ON M6J 1H4, Canada; sonja.elsaid@gmail.com; 2 Institute of Medical Science, Faculty of Medicine, University of Toronto, Toronto, ON M5S 1A8, Canada; stefan.kloiber@camh.ca (S.K.); kim.desmond@camh.ca (K.L.D.); 3 Brain Health Imaging Centre (BHIC), Centre for Addiction and Mental Health, Toronto, ON M5T 1R8, Canada; 4 Department of Biochemistry and Molecular Biology, Johns Hopkins Bloomberg School of Public Health, Johns Hopkins University, Baltimore, MD 21205, USA; rwang155@jh.edu; 5 Department of Psychiatry, Faculty of Medicine, University of Toronto, Toronto, ON M5S 1A8, Canada; 6 Department of Pharmacology and Toxicology, Faculty of Medicine, University of Toronto, Toronto, ON M5S 1A8, Canada; 7 Campbell Family Mental Health Research Institute, Centre for Addiction and Mental Health, Toronto, ON M6J 1H4, Canada; 8 Addictions Division, Centre for Addiction and Mental Health, Toronto, ON M6J 1H4, Canada; 9 Department of Family and Community Medicine, University of Toronto, Toronto, ON M5G 1V7, Canada; 10 Waypoint Research Institute, Waypoint Centre for Mental Health Care, Penetanguishene, ON L9M 1G3, Canada

**Keywords:** social anxiety disorder, pathology, gamma-aminobutyric acid, glutamate, myo-inositol, choline, creatine, N-acetyl aspartate, proton magnetic resonance spectroscopy

## Abstract

Social anxiety disorder (SAD) is characterized by fear and avoidance of social situations. Considering the reduced availability of conventional therapies, we aimed to improve our understanding of the biological mechanisms in SAD by evaluating gamma-aminobutyric acid (GABA) and other neurometabolites (including glutamate + glutamine/glutamix (Glx), N-acetyl aspartate (NAA), myo-inositol (mI), total choline (tCho), and total creatine (tCr) in the dorsomedial prefrontal cortex/anterior cingulate cortex (dmPFC/ACC), dorsolateral prefrontal cortex (dlPFC), and the insula). In this pilot study, we recruited 26 (age: 25.3 ± 5.0 years; 61.5% female) individuals with SAD and 26 (age: 25.1 ± 4.4 years; 61.5% female) sex-age-matched controls. Using proton magnetic resonance spectroscopy, we found that compared to the controls, GABA+ macromolecular signal (GABA+) in dlPFC (t = 2.63; *p* = 0.012) and Glx in the insula (Mann–Whitney U = 178.3; *p* = 0.024) were higher in the participants with SAD. However, no between-group differences were observed in dmPFC/ACC (t = 0.39; *p* = 0.699). Increased GABA+ in dlPFC could be explained by aberrant GABA transporters. In the insula, increased Glx may be associated with the dysfunction of glutamate transporters or decreased activity of glutamic acid decarboxylase in the GABAergic inhibitory neurons. However, these proposed mechanisms need to be further investigated in SAD.

## 1. Introduction

Social anxiety disorder (SAD) is one of the most common anxiety disorders [1,2]. It is characterized by fear and avoidance of social situations and is related to significant functional impairment [3,4]. The prevalence of SAD worldwide is 4.0%, ranging from 0.2% in Nigeria to 12.1% in the US [1,2]. The reported lifetime SAD prevalence in the Canadian population is 14.6% [5]. However, research also indicated that only 1/5 of individuals with SAD seek treatment, and of those, only 30% achieve full recovery [6]. One of the reasons for poor treatment outcomes is that the molecular mechanisms of SAD are not well understood.

Previous research implicated the ‘fear neurocircuitry’ in SAD pathogenesis. This includes the thalamus receiving sensory information and projecting it to the limbic system and various cortical areas [7,8,9]. The amygdala appears to be the key player in the ‘fear neurocircuitry’. It has a role in fear conditioning and emotional processing. In anxiety disorders, the overactivity of the amygdala and insula may lead to ‘misinterpretation’ of stimuli [7,9,10,11].

The dorsal anterior cingulate (dACC) and dorsomedial prefrontal cortex (dmPFC) tend to ‘misinterpret’ sensory signals as threatening. The dorsolateral prefrontal cortex (dlPFC) functions by bringing the ‘threatening information’ to conscious awareness. In contrast, the neural outputs from the rostral anterior cingulate cortex (rACC), ventromedial PFC (vmPFC), and orbitofrontal cortex (OFC) to the amygdala are thought not to provide adequate inhibition, possibly leading to the hyperactivity observed in the amygdala [9,12].

Moreover, the hyperactive amygdala sends circuits to the hippocampus and parahippocampus that provide the current context for aversive stimuli. The caudate and putamen also play a role in the ‘fear neurocircuitry’. The consequences of the impaired circuitry in these brain regions are thought to be associated with avoidant behaviors observed in SAD [13,14,15].

Proton magnetic resonance spectroscopy (^1^H MRS) is an in vivo ionizing-free radiation measurement technique. It provides localized information on concentrations of neurochemicals, which are the byproducts of physiological processes [16]. ^1^H MRS could indicate disturbances in specific brain regions, suggesting aberrant mechanisms in the neurons and glial cells.

Specifically, ^1^H MRS utilizes the magnetic properties of hydrogen nuclei to reveal the molecular structures of various neurochemicals [16,17]. In general, atomic nuclei possess a property called nuclear spin. The spinning nuclei behave like magnets that align with the external magnetic field. When the MRI scanner applies an electromagnetic pulse at a specific radiofrequency (RF), the atomic nuclei absorb its energy, causing them to misalign with the magnetic field and create a detectable MR signal. The absorption of energy is known as resonance, and the specific frequency of resonance depends on the nucleus and its chemical environment [16,17]. Consequently, different types of atoms in a molecule will resonate at different resonant frequencies. The difference in resonant frequencies is known as chemical shift, which designates the connectivity and structure of hydrogen atoms in a molecule. The output of the ^1^H MRS procedure is a spectrum in which the intensity of the MR signal is plotted as a function of resonant frequency to measure the concentration of different protons belonging to various neurochemicals detected in the brain. The resonant frequencies are usually measured in parts per million (ppm) relative to the resonance frequency of protons in a reference compound at the same field strength [16,17].

Among many different neurochemicals, ^1^H MRS has recently been used to study γ-aminobutyric acid (GABA), glutamate (Glu), glutamine (Gln), and glutamate + glutamine (Glx). GABA is a major inhibitory neurotransmitter in the mammalian brain [16,17]. GABA is synthesized by the decarboxylation of Glu by two isozymes of glutamic acid decarboxylase (GAD), GAD 65 and GAD 67 [18,19,20]. Acute and chronic stress were observed to decrease the function of GAD 67 and to impede GABA release from the GABAergic inhibitory neurons [21].

Glutamate (Glu) is the major excitatory neurotransmitter that plays an essential role in cognition, learning, and memory [22]. Glutamine (Gln) is an intermediary metabolite in the Glu–Gln neurotransmitter cycle. Glu and Gln are both markers of neuron–astrocyte integrity [16,20,23,24,25]. In ^1^H MRS, at the lower field strengths (≤3T), Glu and Gln cannot be measured separately. ^1^H MRS concentrations of these two neurometabolites are often reported as glutamix (Glx) [16].

Recently, there has been more interest in investigating the impairments of Glu-Gln and Glu-GABA cycling directly associated with the imbalances in excitatory/inhibitory neurocircuitry. For instance, when GABA is depleted, the activity of GABA on post-synaptic neurons may be insufficient to regulate excitatory currents, leading to hyperactivation of specific brain regions. Previous research on SAD indicated that compared to the healthy controls, downregulated GABA was observed in the thalamus [26] and dmPFC/ACC [27] in those with SAD. However, until now, only two regions involved in the ‘fear neurocircuitry’ have been investigated for levels of GABA in individuals with SAD. Thus, there are significant gaps in research concerning GABA levels in other areas, including dlPFC and the insula.

With these gaps in mind, we designed a study with the primary objective of investigating the levels of GABA in dmPFC/ACC in the general adult population sample with SAD. Our secondary objectives included investigating Glx in dmPFC/ACC, as well as GABA and Glx in dlPFC and the insula. Based on previous research [27], we hypothesized that individuals with SAD would have lower levels of GABA in dmPFC/ACC compared to the healthy controls. Our rationale for conducting the study was to improve our understanding of the pathophysiological mechanisms of SAD and help determine better pharmacological targets for future treatments.

## 2. Results

### 2.1. Demographic and Clinical Participant Characteristics

Table 1 demonstrates the demographic and clinical variables of participants with SAD and their respective healthy controls. No significant differences were found concerning the demographics. The racial backgrounds of participants in the SAD group were 30.8% East Asian, 19.2% South Asian, 42.3% White Caucasian, 3.8% mixed race, and 3.8% other, whereas, in the control group, they were 30.8% East Asian, 30.8% South Asian, 7.7% Black, 23.1% White Caucasian, and 7.7% mixed race (χ^2^ = 5.50; *p* = 0.358). The participants with SAD scored significantly higher on the Liebowitz Social Anxiety Scale (LSAS) and its subscales (Anxiety and Avoidance), Social Interaction Assessment Scale (SIAS), Quick Inventory of Depressive Symptomatology 16 Self-Report (QIDS-16 SR), Sheehan Disability Scale (SDS) total scores, and three SDS domains (Work/School, Social Activities, Family Life/Home Responsibilities). Contrary to these findings, individuals with SAD had significantly lower scores on all four qualities of life domains of the World Health Organization Quality of Life Brief Version (WHOQoL-BREF) scale, including Physical Health, Psychological Health, Social Relationships, and Environmental Health.

### 2.2. Neurochemical Alterations in dmPFC/ACC

#### 2.2.1. Between-Group Neurochemical Comparison

**Figure 1:**
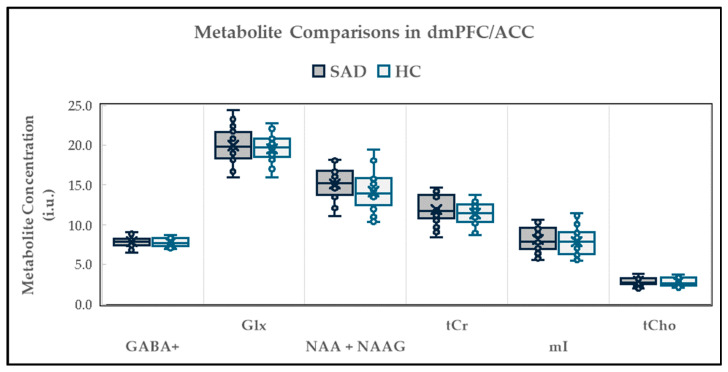
Cross-sectional comparison of dmPFC/ACC metabolites. An independent *t*-test was used to compare the between-group means for GABA+, Glx, NAA + NAAG, tCr, and mI. Mann–Whitney U testing was used to compare means for tCho. dmPFC/ACC = dorsomedial prefrontal cortex/anterior cingulate cortex; SAD = social anxiety disorder; HC = healthy controls; i.u. = institutional units; GABA = gamma-aminobutyric acid; Glx = glutamix; NAA = N-acetyl-aspartate; NAAG = N-acetyl-aspartyl-glutamate; tCr = total creatine; mI = myo-inositol; tCho = total choline. The number of participants (*n*) examined for each metabolite was GABA+ (SAD: 25 vs. HC: 26); Glx (SAD: 24 vs. HC: 24); NAA + NAAG (SAD: 25 vs. HC: 25); tCr (SAD: 26 vs. HC: 24); mI (SAD: 26 vs. HC: 26); tCho (SAD: 24 vs. HC: 24).

The neurochemical levels evaluated between the SAD participants and the healthy controls (HCs) are displayed in Figure 1. The levels of GABA+ were 7.85 ± 0.69 institutional units (i.u.) in the SAD group (*n* = 25) and 7.79 ± 0.55 i.u. in the HCs (*n* = 24) (t = 0.39; *p* = 0.699). For Glx, the levels were 19.94 ± 2.27 i.u. in the SAD participants (*n* = 24), and 19.56 ± 1.68 i.u. in the HC group (*n* = 24) (t = 0.66; *p* = 0.513). For NAA + NAAG, the concentrations were 15.14 ± 1.96 i.u. in the SAD group (*n* = 25) and 13.40 ± 1.65 i.u. for the HCs (*n* = 25) (t = 1.59; *p* = 0.119). For tCr, the levels in the SAD group (*n* = 26) were 11.93 ± 1.71 i.u., and for the HCs (*n* = 24), they were 11.39 ± 1.71 i.u. (t = 1.20; *p* = 0.234).

For mI, the concentrations were 8.13 ± 1.54 i.u. in the SAD (*n* = 26) and 7.88 ± 1.72 i.u. in the HC group (*n* = 26). Mann–Whitney U testing was performed to compare the tCho means between groups, considering that the healthy control group failed the normality test. The levels of tCho were 2.87 ± 0.50 i.u. (*n* = 25) in the SAD group and 2.81 ± 0.52 i.u. (*n* = 24) in the HCs (Mann–Whitney U = 269.5; *p* = 0.542). The results were unaffected when age, sex, voxel proportions of gray matter (GM), and GM/(GM + white matter (WM)) were entered into the ANOVA analysis as covariates.

#### 2.2.2. Relationship Between Neurochemical Levels and Demographic Variables

Supplemental Table S1 shows the Fisher z scores, indicating the relationship between the demographic variables and metabolite levels in dmPFC/ACC. Bonferroni correction was not performed. After accounting for correlation coefficients in the HC group, the only correlation retained was between GABA+ concentrations and income levels, and Glx concentrations and income, where positive correlations were observed.

Supplemental Table S2 shows Pearson’s correlation values between neurochemicals measured in dmPFC/ACC in participants with SAD and the clinical symptoms of avoidant personality disorder (AvPD) and lifetime major depressive disorder (MDD). No significant correlations were found.

#### 2.2.3. Relationship Between Neurochemical Levels and Clinical Variables

Table 2 shows the relationship between dmPFC/ACC metabolite concentrations and clinical variables. Negative correlations were observed between levels of GABA+ and LSAS Anxiety scores, Glx levels and SIAS scores, and NAA + NAAG and LSAS Anxiety scores. Negative correlations were observed between the SDS total scores and tCr, SDS total scores and mI, and SDS total scores and tCho. The SDS Work/School domain scores were also negatively correlated with the same three metabolites above, whereas the SDS Social Life domain total scores were negatively correlated with the levels of tCr. GABA+/Glx was positively correlated with the Social domain on the WHOQofL BREF scale.

#### 2.2.4. Relationship Between Neurochemicals

Supplemental Table S3 shows the relationship between dmPFC/ACC metabolites. The Fisher z scores indicated only a correlation between the concentrations of mI and Glx in the SAD group in dmPFC/ACC.

### 2.3. Neurochemical Alterations in dlPFC

#### 2.3.1. Between-Group Neurochemical Comparison

**Figure 2:**
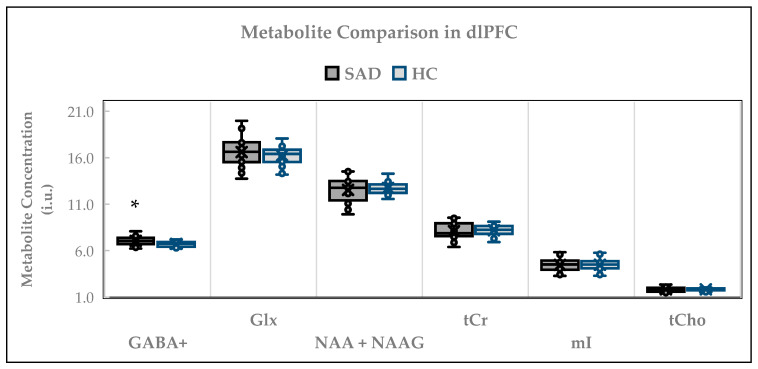
Cross-sectional comparison of dlPFC metabolites. * *p* ≤ 0.05. Independent *t*-test was used to compare all metabolites in dlPFC; dlPFC = dorsolateral prefrontal cortex; SAD = social anxiety disorder; HC = healthy controls; i.u. = institutional units; GABA = gamma-aminobutyric acid; Glx = glutamix; NAA = N-acetyl-aspartate; NAAG = N-acetyl-aspartyl-glutamate; tCr = total creatine; mI = myo-inositol; tCho = total choline. The number of participants (*n*) examined for each metabolite was GABA+ (SAD: 21 vs. HC: 22); Glx (SAD: 22 vs. HC: 22); NAA + NAAG (SAD: 25 vs. HC: 22); tCr (SAD: 24 vs. HC: 23); mI (SAD:24 vs. HC: 25); tCho (SAD: 24 vs. HC: 24).

The neurometabolite levels in dlPFC between SAD and the healthy controls are displayed in Figure 2. The independent *t*-test was used to compare the levels of all metabolites measured in dlPFC. Compared to the HCs (*n* = 22), higher levels of GABA+ (*n* = 21) were detected in the SAD group. GABA+ concentrations in the SAD group were 7.05 ± 0.48 i.u., whereas in the HCs, they were 6.73 ± 0.30 i.u. (t = 2.63; *p* = 0.012). For Glx, the levels in the SAD group (*n* = 22) were 16.60 ± 1.50 i.u., and in the HCs (*n* = 22), they were 16.23 ± 1.08 i.u. (t = 0.94; *p* = 0.354). For mI, the concentrations were 4.47 ± 0.74 i.u. in the SAD group (*n* = 24) and 4.47 ± 0.60 i.u. in the HCs (*n* = 25) (t = −0.02; *p* = 0.985). Similarly, no statistically significant differences were noted when measuring the concentrations of NAA + NAAG, tCho, and tCr. The detected NAA + NAAG concentrations in the SAD group (*n* = 25) were 12.53 ± 1.24 i.u. and 12.74 ± 0.69 in the HCs (*n* = 22) (t = −0.72; *p* = 0.479), whereas the tCr concentrations were 8.09 ± 0.88 i.u. in the SAD group (*n* = 24) and 8.19 ± 0.57 i.u. in the HCs (*n* = 23) (t = −0.48; *p* = 0.634). For tCho, the mean concentration was 1.83 ± 0.27 i.u. in the SAD group (*n* = 24) and 1.83 ± 0.14 i.u. in the healthy controls (*n* = 24) (t = −0.04; *p* = 0.973). The results were unaffected when age, sex, GM, and GM/(GM + WM) were separately entered as covariates into the ANOVA analysis.

#### 2.3.2. Relationship Between Neurochemical Levels and Demographic Variables

Supplemental Table S4 shows the Fisher z scores, indicating the relationship between the demographic variables and metabolite levels in dlPFC. Bonferroni correction was not performed. The statistically significant correlations were observed between GABA+ concentrations and income levels, tCr concentrations and level of education, and mI levels and sex, where higher mI levels were recorded for male subjects with SAD.

Supplemental Table S5 shows the Pearson’s correlation values between the neurometabolites measured in dlPFC for the participants with SAD, who also had clinical symptoms of AvPD and lifetime MDD. Significant correlations were found between the levels of GABA+ and the clinical symptoms of AvPD, GABA+ levels, and lifetime MDD, and between the concentrations of mI and the clinical symptoms of AvPD. The concentration differences of the neurometabolites mentioned above were not affected when AvPD and MDD were separately entered into the ANOVA analysis as covariates.

#### 2.3.3. Relationship Between Neurochemical Levels and Clinical Variables

Table 3 shows the relationship between dlPFC metabolites and clinical variables. Negative correlations were observed between Glx concentrations and the WHOQoL-BREF Social scores, the levels of NAA + NAAG and SIAS total scores, tCr and the WHOQoL-BREF Social scores, mI levels and the WHOQoL-BREF Psychological scores, and mI levels and the WHOQoL-BREF Social scores. A positive correlation was observed between tCho concentrations and the WHOQoL-BREF Physical scores.

#### 2.3.4. Relationship Between Neurochemicals

Supplemental Table S6 shows the Fisher z scores between the metabolites in dlPFC. Significant correlations were observed between GABA+ and tCr and GABA+ and mI concentrations. The levels of Glx were positively correlated to NAA + NAAG levels, whereas NAA + NAAG concentrations were correlated to tCr. tCho levels were positively correlated with tCr and mI concentrations.

### 2.4. Neurochemical Alterations in Insula

#### 2.4.1. Between-Group Neurochemical Comparison

**Figure 3:**
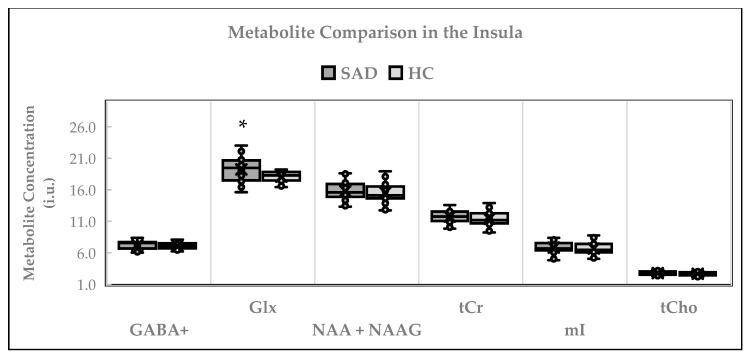
Cross-sectional comparison of metabolites in the insula. * *p* ≤ 0.05. The independent *t*-test was used to compare the means for GABA+, NAA + NAAG, Cr, mI, and tCho. Mann–Whitney U testing was used for Glx. i.u. = institutional units; SAD = social anxiety disorder; HC = healthy controls; GABA = gamma-aminobutyric acid; Glx = glutamix; NAA = N-acetyl-aspartate; NAAG = N-acetyl-aspartyl-glutamate; tCr = total creatine; mI = myo-inositol; tCho = total choline. The number of participants (*n*) examined for each metabolite was GABA+ (SAD: 22 vs. HC: 26); Glx (SAD: 24 vs. HC: 24); NAA + NAAG (SAD: 23 vs. HC: 26); tCr (SAD: 24 vs. HC: 26); mI (SAD: 24 vs. HC: 26); tCho (SAD: 24 vs. HC: 23).

Figure 3 shows the cross-sectional comparison between the metabolites in participants with SAD and the HCs. An independent *t*-test was used to compare the levels of GABA+, NAA + NAAG, tCr, mI, and tCho between the participants with SAD and the HCs. The Mann–Whitney U test was used to compare Glx means, considering that the HC group failed the test of normality (Shapiro–Wilk test statistic: 0.914; *p* = 0.044). Compared to the HCs (*n* = 24), higher levels of Glx (*n* = 24) were detected in the SAD group. Glx concentrations in the SAD group were 19.22 ± 1.88 i.u., whereas in the HCs, they were 18.08 ± 0.90 i.u. (statistic = 178.5; *p* = 0.024). The GABA+ levels in the SAD group (*n* = 22) were 7.25 ± 0.65 i.u. in the HCs (*n* = 22), they were 7.25 ± 0.65 i.u. (t = 0.94; *p* = 0.354).

No significant differences were found between the groups in terms of the concentrations of other metabolites. The mean concentrations were 15.80 ± 1.41 i.u. in the SAD group (*n* = 23) and 15.56 ± 1.56 i.u. in the HCs (*n* = 26) (t = 0.58; *p* = 0.566) for NAA + NAAG; 6.83 ± 0.93 i.u. in the SAD group (*n* = 24) and 6.67 ± 1.06 i.u. in the HCs (*n* = 26) (t = 0.76; *p* = 0.577) for mI, 11.74 ± 1.02 i.u. in the SAD group (*n* = 24) and 11.39 ± 1.15 i.u. in the HCs (*n* = 26) (t = 0.72; *p* = 0.577) for tCr, and 2.81 ± 0.34 i.u. in the SAD group (*n* = 24) and 2.67 ± 0.31 i.u. in the HCs (*n* = 23) (t = 0.80; *p* = 0.141) for tCho. The results were unaffected when age, sex, GM, and GM/(GM + WM) were entered into the ANOVA analysis as covariates.

#### 2.4.2. Relationship Between Neurochemical Levels and Demographic Variables

Supplemental Table S7 shows the Fisher z scores, indicating the relationship between the demographic variables and the metabolite levels in the insula. Bonferroni correction was not performed. A statistically significant correlation was only noted between GABA+ concentrations and the level of education. Supplemental Table S8 indicates Pearson’s correlation coefficients between metabolite concentrations and AvPD and between the metabolite concentrations and the lifetime MDD. No correlations were observed concerning these comparisons.

#### 2.4.3. Relationship Between Neurochemical Levels and Clinical Variables

Table 4 shows the relationship between metabolite concentrations in the insula and the clinical variables. A negative correlation was observed between tCr levels and LSAS Anxiety scores, while a positive correlation was noted between levels of mI and the WHOQoL BREF, Environmental scores.

#### 2.4.4. Relationship Between Neurochemicals

Supplemental Table S9 shows the Fisher z scores between the metabolite concentrations in the insula. The only significant correlation was shown between GABA+ and Glx concentrations.

### 2.5. Spectroscopy Quality Measures

#### 2.5.1. GABA+ and Glx Quality Measures

GABA+ and Glx quality measures were compared between the two groups to demonstrate the reliability of our concentration measurements. The GABA+ and Glx quality measures are shown in Supplemental Table S10. They included GABA+ signal-to-noise ratios (SNRs), GABA+ full-width half maximum (FWHM), GABA+ fit error percentage (%), GABA+/water (H_2_O) fit error%, Glx SNR, Glx FWHM, Glx fit error%, Glx/H_2_O fit error%, and H_2_O FWHM.

No statistically significant differences were observed concerning any quality measures, except for GABA+ FWHM, GABA+ fit error%, and GABA+/H_2_O fit error% in dlPFC, in which these three parameters were higher in the SAD group than in the HCs. The differences between these GABA+ quality parameters indicate that the GABA+ concentration differences in dlPFC need to be carefully interpreted.

#### 2.5.2. NAA + NAAG, tCr, mI, and tCho Quality Measures

To demonstrate the reliability of our measures of the NAA + NAAG, tCr, mI, and tCho concentrations, the quality measures that included NAA + NAAG Cramer-Rao Lower Bounds (CRLB)%, tCr CRLB%, mI CRLB%, and tCho CRLB%, H_2_O SNR, H_2_O FWHM, and H_2_O frequency drifts were evaluated for all three ROIs. No statistically significant differences between the SAD and HC groups were observed for the quality measures. Moreover, no substantial changes were noted between the groups when comparing the GM, WM, CSF, and GM/(GM + WM) ratios in all three regions. Supplemental Table S11 compares the quality measures of the spectroscopic parameters indicated above.

## 3. Discussion

The present study investigated the neurometabolite levels in a small sample of individuals with SAD and a group of sex-age-matched healthy controls in the three brain regions involved in the ‘fear neurocircuitry’ (dmPFC/ACC, dlPFC, and the insula). The purpose was to understand the related molecular pathophysiological mechanisms in SAD that would ultimately lead to the development of better SAD treatments. Below, we discuss the findings for each region of interest.

### 3.1. dmPFC/ACC

Contrary to our predictions, no differences in Glx (glutamate + glutamine) or GABA+ concentrations were found between the two groups in dmPFC/ACC. Our data is aligned with another study, demonstrating no differences between the two groups in Glx concentrations in ACC [28]. Yet, our results on GABA+ contrasted with the findings of lower GABA+ in the SAD group in dmPFC/ACC [27]. However, the correlational analysis showed reductions in both GABA+ and Glx levels, as the severity of social anxiety increased. The drop in the concentrations of both neurotransmitters with increasing symptoms of SAD may point to a form of pathology resembling frontotemporal degeneration [29,30].

The concentrations of both mI and Glx decreased with increasing severity of SAD. Furthermore, we noted a positive correlation between the concentrations of Glx and mI. The parallel decrease in these two metabolites (designated by the positive correlation) may indicate glial cell damage. Considering that mI is a marker of cell membrane integrity and Glu is converted to Gln and stored in astrocytes, the observed decreases in mI may have been a product of astrocyte cell membrane perturbations, potentially affecting the export of Gln and its precursor availability to Glu in the presynaptic neurons [31,32].

The observed decreases in NAA + NAAG, tCr, mI, and tCho concentrations with increasing SAD severity and functional disability further support the neurodegenerative-like mechanistic changes in dmPFC/ACC in individuals with SAD [29,30]. The levels of NAA + NAAG decreased with increasing LSAS scores, and so did the levels of tCr with increasing SDS scores. It was previously shown that lower-than-normal levels of NAA + NAAG designated neuronal loss [33,34], while lower NAA + NAAG and tCr were also linked to mitochondrial dysfunctions and impairments in energy metabolism [16,33]. Moreover, previous research showed that deficiencies in NAA and tCr were linked to anxiety states in animal and human studies. Thus, the impairments in the mitochondria and the downregulations in the ATP production may have eventually resulted in neuronal and glial cell death and tissue degeneration in the SAD group [35,36].

Decreased concentrations of mI and tCho were also noted with increasing functional disability in participants with SAD in dmPFC/ACC. Reduced mI was previously linked to glial cell loss [37,38]. Myo-inositol and tCho concentration reductions may have indicated the presence of the mechanisms involved in cellular membrane repair, as cell membrane repair is the process necessary to ameliorate neuronal and glial cell damage [39,40].

Another mechanism, the downregulated function and the impairments of G-coupled Protein Receptors (GCPRs), may have also explained the reduced levels of mI, with increases in functional disability in participants with SAD. mI is the byproduct of the second messenger, inositol 1,4,5-triphosphate (IP_3_), which is normally released when GCPRs are activated [41,42,43,44,45]. As such, decreased concentrations of neurotransmitters, previously shown to be reduced in SAD (such as dopamine and serotonin) that normally bind to GCPRs, may have led to reductions in mI [43,46].

In summary, with increasing SAD severity, the reduced levels of GABA+, Glx, NAA + NAAG, tCr, tCho, and mI may have indicated glutamatergic, GABAergic, and glial cell damage, involving the mechanisms of mitochondrial and cellular damage, decreased ATP production, and the downregulation of GCPRs and second messenger systems. Nevertheless, considering these pathological mechanisms in SAD are still poorly understood, more research is needed to fully elucidate the relationship between the spectroscopic metabolites and their implicated tissue impairments.

### 3.2. dlPFC

The data for dlPFC indicated elevated GABA+ concentrations in the individuals with SAD compared to the healthy controls, while no other neurochemical changes were observed. Elevated GABA+ with increasing severity of social anxiety symptoms in dlPFC was demonstrated in one other study assessing adolescent and early adult females [47]; however, no other cross-sectional studies in SAD have been identified in the medical literature. In addition, the research showed that GABA+ was elevated in dlPFC in individuals with post-traumatic stress disorder (PTSD) in another study [48].

dlPFC has a role in the processing of working memory and the cognitive control of the limbic system, including the amygdala [49,50,51,52]. The over-inhibition of dlPFC may have occurred due to the decoupling between dlPFC and the amygdala. The weaker connectivity in the circuits between dlPFC and the amygdala was previously observed in specific phobia (SP) [53], PTSD [54], and acute stress [55]. The overinhibited dlPFC may have led to decreases in the top-bottom control of the amygdala and its observed overactivity in emotional processing. However, these processes still need to be demonstrated in SAD.

One possible explanation for the elevated GABA+ is the reduced activity of GABA transporters, such as GAT 1-3 (responsible for the reuptake of GABA from the synaptic cleft back into astrocytes). Previous research revealed that the selective blocker of GAT-1, tiagabine, increased the levels of GABA in the synaptic cleft in individuals with anxiety [56], suggesting that the impaired functioning of GAT may have resulted in GABA+ increases. Moreover, several SNP variants of the solute carrier family 6-member 1 (SLC6A1) gene, encoding for GAT-1, were linked to anxiety disorders [57]. However, the specific functioning of GAT and its polymorphic variants has not been explicitly studied in SAD, warranting further investigation.

Interestingly, positive correlations between GABA+ and tCr, as well as GABA+ and mI, concentrations were noted in dlPFC. tCr was upregulated with decreasing scores in social quality of life, while mI level increases with decreases in psychological and social quality of life scores were also observed. The upregulation of tCr concentrations may have indicated the increased cellular need for ATP [33,58,59]. For example, previous research has demonstrated that in individuals infected with HIV, increased tCr was linked to active gliosis (the state during which neuroinflammatory processes are mobilized), aiming to decrease the effects of glial-cell viral infections. Under these conditions, extra ATP production is needed to aid the cellular regenerative processes [58,59]. Thus, the increased tCr in dlPFC may have indicated the presence of gliosis in the astrocytes. Moreover, the positive correlation between GABA+ and tCr concentrations may have indicated damage to GAT in the astrocytes associated with the mechanisms of gliosis. The positive correlation between the levels of mI and GABA+ could be explained by the damage to the same cellular targets in the phospholipid bilayer of astrocytes, given that perturbations of GAT located in the phospholipid bilayer ultimately led to upregulated GABA+, and the release and the increase of mI also may have resulted from the breakdown of phosphoinositol metabolites in the cellular membrane. Furthermore, damage to the phospholipid bilayer of astrocytes was implied by the positive correlation between tCho and mI and tCho and tCr concentrations.

However, it is worth mentioning that aside from its role in regulating the energy metabolism in the brain, creatine has an essential role in regulating E/I neurocircuitry [60]. For example, creatine was demonstrated to enhance the density of GABA neurons in cell cultures [61], reverse the damage to GABAergic interneurons [62], and stimulate Glu reuptake by GABAergic cells, so that Glu could be converted to GABA by glutamic acid decarboxylase (GAD). By influencing GABAergic interneurons, creatine indirectly downregulated the excitatory currents generated by extracellular Glu [63,64,65]. Consequently, the elevated GABA+ levels may have resulted from the increased activity of GAD in the GABAergic interneurons, leading to the observed positive correlation between GABA+ and tCr levels.

Nevertheless, the sources of elevated GABA+ in dlPFC in the SAD group need to be investigated further. The proposed GAT disturbances should specifically be studied in dlPFC between individuals with SAD and HCs using positron emission tomography (PET), whereas PET or electron microscopy [66] could be utilized to investigate the functioning of GABAergic neurons.

Furthermore, the decreased levels of NAA + NAAG with increasing situational social anxiety scores and the positive correlation between the levels of Glx and NAA + NAAG may indicate astrocyte disturbances. NAA + NAAG serves as a reservoir for Glu. It is converted to Glu in the astrocytes, on demand [67]. Consequently, the dependence of Glu on NAA + NAAG resources would have explained the positive correlation between the two neurochemicals in SAD. On its own, downregulated NAA + NAAG implicates mitochondrial dysfunction in dlPFC.

In summary, our investigations of dlPFC in SAD point to several mechanisms, including downregulated mitochondrial activity, gliosis of astrocytes accompanied by an increased ATP demand, and disturbances to the astrocyte phospholipid bilayer. With the increase in GABA+ concentrations, several pathological mechanisms were suspected, such as disturbances of GAT. However, these mechanisms need to be further investigated.

### 3.3. Insula

To our knowledge, this study is the first to report on GABA+/Glx changes in the insula in SAD. Compared to the healthy controls, increased levels of Glx were observed in the participants with SAD, while no changes were noted concerning other spectroscopic metabolites. The increase in Glx may indicate higher-than-normal excitatory currents in the insula since Glx contains Glu, which is the major excitatory neurotransmitter. Coincidentally, several fMRI studies on the insula in SAD indicated the hyperactivity associated with enhanced threat processing in response to receiving social information, such as viewing facial expressions [51].

A marginal decrease in GABA+ levels relative to the increasing symptoms of social anxiety, and a positive correlation between GABA+ and Glx concentrations were observed in the SAD group, pointing to the mechanistic disturbances in GABA/Glu/Gln cycling.

Two possible pathological mechanisms may have contributed to the observed changes in GABA+ and Glx. Increased Glx and marginally decreased GABA+ levels may have resulted from the impairment/reduced activity of the excitatory amino acid transporters (EAATs), which sequester Glu from the glutamatergic synapse back into astrocytes for storage. There are five types of EAAT, of which EAAT 1, 2, and 3 are expressed in human astrocytes [68,69]. The impairments in EAAT transporters may have led to the accumulation of Glu at the synapse and the observed higher-than-normal levels of Glx in the insula.

Moreover, the sources of Glu in the astrocytes also come from GABA, sequestered from the synapse; thus, the shift in resourcing Glu from GABA, rather than from the recycled synaptic Glu, may potentially explain the appearance of the positive correlation between GABA+ and Glx levels in the insula [20,70,71].

Interestingly, previous research reported a correlation between the severity of the symptoms in individuals with obsessive-compulsive disorder and the mutation of the EAAT 3, solute carrier family 1 member 1 (SLC1A1) gene [72]. While there is no direct evidence of such mutations occurring in SAD, a study investigating gene–environment interactions on adolescents with social anxiety [73] demonstrated that punitive control parenting together with the polymorphic variants of SLC1A1 was more likely to be found in adolescents with social anxiety [73].

The second proposed mechanism, which may explain the elevated Glx in the insula, is impaired GAD, an enzyme that converts Glu into GABA, making GABA ready to be released into the glutamatergic synapse [20,70,71]. GAD exists in two isoforms, GAD65, encoded by the GAD2 gene, and GAD67, encoded by GAD1 [60,74]. Although not exclusively studied in SAD, the evidence supporting the notion that impairments of GAD may be involved comes from previous studies showing that certain polymorphic variants of GAD1 were associated with an increased risk for developing anxiety disorders (including SAD) [75]. A single-nucleotide polymorphism (SNP) variant of GAD2 was also identified as posing a risk for developing anxiety [76]. However, the two proposed mechanisms described above need to be further investigated.

The observed decreases in tCr with increasing anxiety may indicate aberrant mitochondrial function and energy metabolism [77,78], while downregulations in mI may represent astrocyte cell membrane disturbances [37,38]. These mechanisms were explained in the previous section.

In short, our investigations of the insula indicate neurochemical imbalances pointing to impairments in mitochondria, energy metabolism, and astrocyte cell membranes. Moreover, we proposed two pathological mechanisms, which may explain our observations of elevated Glx and marginal decreases in GABA+. The two proposed mechanisms include decreased activity of EAAT in the glial cells and downregulated GAD in the GABAergic inhibitory interneurons. However, future studies should specifically address the proposed pathological changes.

### 3.4. Study Limitations

There are several limitations in our study. First, we used a 3T MRI scanner, which did not provide sufficiently high spectral resolution to measure metabolites with similar molecular structures [16] separately. As a result, we could not measure NAA, NAAG, Glu, and Gln individually. A separate assessment of Glu and Gln levels in SAD would have given us a better view of GABA+/Glu/Gln cycling. Moreover, considering that there are molecular states in which Glu and Gln levels may be altered in opposite directions, the separate measurements of Glu and Gln would have better identified the regional impairments [69]. Thus, future studies should investigate metabolic disturbances in SAD using higher magnetic fields (≥7T). Utilizing this MRI technology would also improve the quantification of all metabolites [16].

Second, Glu has many roles in the brain beyond excitatory neurotransmission. Given that spectroscopy measures both the intra- and extracellular Glu in ROIs, it was challenging to distinguish between Glu released at the glutamatergic synapse and the intracellular Glu [31,69]. Although measurements of ^1^H MRS metabolites could imply the molecular mechanisms associated with certain disease states, ^1^H MRS does not directly measure neurotransmission.

Third, we conducted a pilot study with 26 participants per group, which allowed us to fulfill our primary purpose of comparing various metabolite concentrations between the groups. Due to the small sample size and exploratory nature of our study, it is possible that an imbalance between groups on non-controlled demographics could result in unanticipated biases. However, larger sample sizes are needed to conduct a broader correlational analysis, which could assess the correlations between multiple demographics, clinical measures, and spectroscopic metabolite levels. Furthermore, as determined by the SAD severity measures (total average LSAS scores), our sample had moderate-to-severe SAD; thus, our data comparing metabolite levels reflects the mechanistic differences between individuals with moderate-to-severe SAD and healthy controls.

Fourth, while the relatively good quality of the concentration measurements was demonstrated in our study, we observed significant variations between the two groups in the measurements of GABA+ FWHM, GABA+ fit error%, and GABA/H_2_O fit error% in dlPFC. FWHM measures spectral resolution, affected by inhomogeneities of the scanner’s magnetic field, poor shimming procedures, and eddy currents. Consequently, the variations in any of those measures may have contributed to the observed differences in FWHM [79,80]. Fit error% indicates the differences between spectral peaks produced by the actual data and the modeled peaks from the software [81]. Increased deviations between the actual data and the modeled peaks may have varied between the SAD group and the HCs in dlPFC. As a result, our measures of GABA+ concentrations in dlPFC should be carefully interpreted and reproduced in future studies.

Fifth, we conducted multiple correlational comparisons of metabolites with demographic and clinical outcome measures, which may have resulted in type I errors (false-positive results). However, we mindfully avoided performing statistical corrections for multiple comparisons to prevent making type II errors (false negative results). Presuming that the correlated outcome measures shared the same degree of variance, type I errors were less likely to occur when conducting multiple statistical comparisons on the same dataset [82]. Nevertheless, despite the abovementioned limitations, our research provides valuable information on spectroscopic metabolic changes in the three brain regions implicated in SAD.

## 4. Materials and Methods

This was a cross-sectional pilot study in which individuals with and without SAD were enrolled, screened, assessed using several outcome measures, and scanned with an MRI machine to measure the levels of neurochemicals in dmPFC/ACC, dlPFC, and the insula.

### 4.1. Participants

The study recruited 26 individuals with moderate-to-severe SAD and 26 healthy controls to meet the required power of Cohen’s d of 0.8, α = 0.05, and 1–β = 0.8 (80% power), assuming a 30% decrease in GABA+ values in dmPFC/ACC in the SAD group. The SAD group enrolled in the study constituted male and female participants (18 years of age and over) who met the criteria for current SAD according to the Structured Clinical Interview for Diagnostic Statistical Manual Research Version–5 Disorders (SCID-RV for DSM-5) [83] and obtained a total score of ≥60 on the Liebowitz Social Anxiety Scale (LSAS) [84]. Participants with a history of any current or past psychiatric illnesses or personality disorders (as per SCID-RV and PD) [83,85], except for mild current major depressive episodes, persistent depressive disorder, any other anxiety disorder, or any cluster C personality disorder, were excluded from the study.

Healthy control subjects were sex-age-matched to the participants in the SAD group. They were generally healthy, scored ≤ 30 on the Liebowitz Social Anxiety Scale (LSAS) [84], and did not meet the criteria for any psychiatric or personality disorder as per SCID-RV or SCID-PD [83,85].

Moreover, the exclusion criteria that applied to both groups were current or past unstable medical conditions, brain injury or head trauma, suicidal ideation, pregnant or lactating women, those using regular pharmacological treatments with psychotropic medication, using cannabis products or smoking cigarettes, or individuals with a positive urine test for any illicit substances or psychotropic medications. Considering that the study procedures included measuring neurochemicals with MRI in the brain, claustrophobic participants, those who could not lie down for at least 1 h without movement, and/or subjects having metal objects in the body or implanted electronic devices, including cochlear implants, metal fragments in the eye or brain, pacemakers, or ferromagnetic aneurysm clips were also excluded. All participants were instructed not to drink alcoholic beverages and limit their coffee intake to <200 mg/day of caffeine (approximately equal to a daily intake of one cup of brewed coffee or two cups of caffeinated tea or instant coffee) within 1 week of their scanning procedure.

This study was approved by the Research Ethics Board of the Centre for Addiction and Mental Health (CAMH), Toronto, Ontario, Canada (Ethics # 100-2019), on 4 November 2019 and renewed until 1 November 2024. All participants provided written informed consent before enrolling in the study. All procedures involving human participants were performed following the ethical standards of the institutional research committee and with the 1964 Helsinki Declaration and its later amendments and comparable ethical standards.

### 4.2. Study Procedures

Study participants were recruited from clinical sites at CAMH by posting study flyers at the University of Toronto, in the community, and by advertising online using Facebook, Instagram, and Kijiji. Study ads were also sent to students at the University of Toronto through the listservs. Participants who had previously consented to be notified about future studies were also recruited. Initially, a short pre-screening interview was conducted over the phone, assessing the general eligibility criteria. The interested participants who met the pre-screening criteria were invited to participate in three separate sessions after obtaining verbal informed consent for study participation.

The first session was a screening interview conducted using WebEx technology. During the interview, the general eligibility criteria were assessed using SCID-RV [83] and SCID-PD [85], LSAS [84], Medical and Psychiatric History Form, Sociodemographic Assessment Form, and Medication Log. Participants who met the criteria during the online screening interview were invited to attend the second part of the screening visit, which included providing a urine sample at the medical laboratory at CAMH. The urine test included screening for the consumption of cannabis products and illicit substances (e.g., cocaine, heroin, amphetamines, opioids, and hallucinogens) and the consumption of psychotropic and pain medications. Female participants were screened for pregnancy. Participants whose urine tests were negative were invited to the study visit.

The study visit consisted of two parts. During the first session, several measures were administered, including SIAS [86]; QIDS 16-SR [87], SDS [88], and WHOQoL-BREF [89]. LSAS [84] was repeated during the study visit to ensure all subjects still met the cut-off eligibility criteria. Participants were also asked if they had started or discontinued any medications since the screening session. For the subjects’ safety, urine pregnancy dipstick and urine cup toxicology tests were conducted to ensure that no female subjects were pregnant or that any participants were inebriated during the MRI scan. Moreover, a short physical examination assessed the participants’ height, weight, and blood pressure. Alcohol and caffeine consumption were also recorded for the week before the MRI test.

The second part of the study visit consisted of the MRI scan, which lasted approximately 1 h. During this test, the concentrations of spectroscopic neurochemicals were quantified in dmPFC/ACC, dlPFC, and the insula using ^1^H MRS procedures.

### 4.3. Outcome Measures

SCID-RV [83] and SCID-PD [85] are semi-structured interview guides. The purpose of these questionnaires is to make diagnoses according to the American Psychiatric Association’s Diagnostic and Statistical Manual for Mental Disorders (DSM) criteria [3]. A socio-demographic assessment determined the demographic characteristics of study participants, including their age, sex, gender, highest level of education, employment status, income, marital status, racial and ethnic background, housing, and living arrangements.

The Medical/Psychiatric History Form is a checklist of major physical illnesses and acute or chronic medical conditions, including neurological disorders (e.g., stroke, epilepsy, and seizures), endocrine (diabetes), autoimmune (e.g., rheumatoid, psoriatic arthritis, or lupus), and chronic viral infections (e.g., HIV and hepatitis). The Psychiatric History Form records previous clinical diagnoses of mental health conditions and the presence of current psychotic, post-traumatic symptoms, and suicidal thoughts. The Medication Log records the type of medications/natural health products used, their dose and frequency of use, and when the product use was initiated and stopped.

LSAS [84] is a 24-item questionnaire designed to assess several social interactions and performance situations feared by those with SAD. The scale is divided into two subscales, Fear and Avoidance. The 24 items are rated on a Likert Scale from 0 to 3 for fear and avoidance in specific situations. The maximum score is 144. The occurrence of SAD is unlikely at a cut-off score of 30 and below. The LSAS total scores point to the severity of SAD. Accordingly, scores of 30–60 indicate mild SAD, 60–90 moderate SAD, and >90 severe SAD. The LSAS scale was demonstrated to have an internal consistency of Cronbach’s α = 0.95, test-retest reliability of 0.83, and robust convergent validity [90].

SIAS [86] is a 20-item Likert scale that assesses two situations: the fear of interacting in social situations and being observed by others. Once completed, all 20 items are summed up to produce a total score. The possible scores range from 0 to 80. Higher scores are associated with higher social anxiety. Scores of 36 or higher indicate the presence of SAD. The SIAS has been demonstrated to have an internal consistency of Cronbach’s α = 0.93 when tested on a sample of individuals with SAD. A 12-week test-retest reliability was 0.92, and the construct validity was 0.72 [86] when the scale was compared with the Social Phobia Scale [91].

QIDS 16-SR [87] is a 16-item inventory designed to rate the severity of depression. It assesses the 9 criteria used for diagnosing a major depressive disorder. The total scores range from 0 to 27. Higher scores are associated with increased severity of depression. The absence of depression is determined by the scores 0–5, mild depression by 6–10, moderate depression by 11–15, severe depression by 16–20, and very severe depression by 21–27 scores. QIDS-SR demonstrated a high internal consistency (Cronbach’s α = 0.86). It was highly correlated to the Hamilton Depression Scale (HAM-D 24) (0.86) [87], and it had a test-retest reliability of 0.49–0.77 over 2 weeks [92].

SDS [88] is a 5-item questionnaire measuring the extent to which a disability/illness/health problem interferes with work/school, social life/leisure activities, and family life/home responsibilities. Each domain on the SDS is rated on a scale of 0 to 10 (0 being unimpaired and 10 being highly impaired). Scores of 5 or above on each subscale indicate a significant functional impairment. The internal consistency of SDS was moderate (Cronbach’s α = 0.55), while the validity was 0.7 when the scale was compared to the Liebowitz Self-Rated Disability Scale (LSRDS) [93].

The World Health Organization Quality of Life Brief Version (WHOQofL-BREF) [89] is a shorter version of the full-scale World Health Organization Quality of Life. Instead of 100, the WHOQofL BREF scale contains 26 items assessing the quality of life. The scale consists of four domains. They include physical, psychological, environmental health, and social relationships. The items are scored using a scale from 1 to 5, and the scores are transformed to the 0–100 scale. The internal consistency was Cronbach’s α = 0.82 for physical health, 0.81 for psychological, 0.80 for environmental health, and 0.68 for social relationships. Construct validity was assessed by correlating domain scores with each general item. The scores ranged from 0.48–0.70 for physical health, 0.50–0.65 for psychological health, 0.45–0.57 for social relationships, and 0.47–0.56 for environmental health [94].

### 4.4. Scanning Procedures and Spectroscopic Parameters

#### 4.4.1. Scanning Procedures

The study participants were scanned using a 3T General Electric (GE) MR 750 scanner (General Electric, Waukesha, WI, USA), equipped with a 32-channel head coil (Nova Medical Inc., Wilmington, MA, USA). High-resolution T1 weighted images were acquired using 3D IR-prep fast spoiled gradient (FSPGR) sequence brain volume (BRAVO) (TE = 3.0 ms, TR = 6.7 ms, inversion time (TI) = 650 ms., flip angle = 8°, resolution = 0.9 mm isotropic, and scan time = 5 min). CHESS (Chemical Shift Selective Saturation) was used for water suppression [95]. MRS spectra (sequence details below) were obtained from the three regions of interest (ROIs): dmPFC/ACC, dlPFC, and the insula.

**Figure 4:**
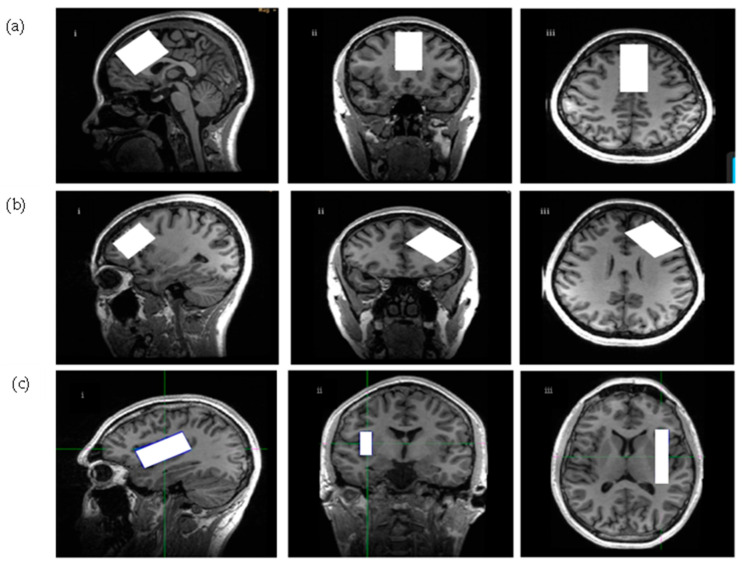
Voxel placements for (**a**) dmPFC/ACC, (**b**) dlPFC, and (**c**) insula. For dmPFC/ACC, the voxel was placed 4 cm in the anterior-posterior, 2 cm right-left, and 2 cm in the superior-inferior direction (total voxel volume was 24 cm^3^). For dlPFC, the placements were 3 cm in the anterior-posterior, 3 cm right-left, and 2 cm in the superior-inferior direction (total voxel volume was 18 cm^3^). For the insula, the voxel was placed 5.5 cm in the anterior-posterior direction, 1.2 cm right-left, and 2.5 cm in the superior-inferior direction. The voxel placement is presented in the (**i**) sagittal, (**ii**) axial, and (**iii**) coronal views. The white rectangles indicate voxel placements. dmPFC/ACC = dorsomedial prefrontal cortex/anterior cingulate cortex; dlPFC = dorsolateral prefrontal cortex.

dmPFC/ACC voxel was placed on a reformatted oblique axial image, parallel to ACC and the corpus callosum on the sagittal plane. The voxel was kept away from the corpus callosum, the skull, the anterior frontal lobe, and the peri-genial cingulate cortex. The voxel size was 4 cm (anterior-posterior), 2 cm (right-left), 3 cm (superior-inferior), and 24 cm^3^. The dlPFC voxel was placed on a double oblique image between the superior and inferior frontal gyrus. The dlPFC voxel size was 18 cm^3^: 3 cm (anterior-posterior), 3 cm (right-left), 2 cm (superior-inferior). A voxel over the right insula was initially reformatted from the sagittal BRAVO T1-weighted scan of the axial and coronal images. Then, a voxel was placed on an oblique axial image, parallel to the lateral/Sylvian fissure, while ensuring that it stayed on the sagittal plane above the temporal lobe. The voxel was kept from the caudate and the temporal gyrus on the axial and coronal planes. The insula voxel extended 1.2 cm (right-left), 5.5 cm (anterior-posterior), and 2.5 cm (superior-inferior). The total volume was 16.5 cm^3^. The voxel placements for dmPFC/ACC, dlPFC, and the insula are shown in Figure 4.

#### 4.4.2. Spectroscopic Parameters

Mescher–Garwood Point Resolved Spectroscopy (MEGA-PRESS) [96] was used to acquire ^1^H MRS data. The sequence parameters are outlined in Table 5. MEGA-PRESS uses J-difference editing, which acquires spectra under two different conditions (editing-ON and editing-OFF) throughout a scan in an interleaved manner. The editing pulses are radio frequency (RF) pulses with a pulse width of 14.4 msec. During the editing-ON acquisition, the editing pulse is placed at 1.9 ppm to invert GABA-H3 spins, refocusing the evolution of J-coupled GABA-H4 spins at 3.0 ppm. During the editing-OFF acquisition, the editing pulse is placed at 7.5 ppm, a region with no metabolite signatures. The difference spectrum results from subtracting the two spectral acquisitions, which uncovers the GABA peak at 3.0 ppm [81,96,97,98]. However, the peak also contains a macromolecular (MM) signal. The MMs resonate at 1.7 ppm, near the editing-ON pulse; thus, MMs are coedited with GABA, producing a peak at 3.0 ppm in the difference spectrum. Given that the GABA-edited peak is contaminated with MMs, we refer to the measurement of GABA as GABA+. Water-unsuppressed reference spectra were used for internal tissue/water referencing. These water-unsuppressed reference spectra were acquired before the water suppression procedures [81]. The AUTOSHIM (manufacturer’s automated shimming) procedures were conducted before each scan to ensure the full width at half maximum (FWHM) was 12 Hz or less.

### 4.5. Spectroscopic Data Processing

GABA+ and Glx peaks were fitted on the difference spectrum and quantified with Gannet 3.1.5 [81]. Gannet and SPM12 (www.fil.ion.ucl.ac.uk/spm, accessed on 1 December 2023) were used for the voxel-to-T1-weighted image registrations. GABA+ and Glx were reported in institutional units (i.u.), whereby the water signal from the MEGA-PRESS acquisition without spectral editing was used as an internal water reference. The MEGA-PRESS editing-OFF data evaluating concentrations of NAA + NAAG, tCr, tCho, and mI were analyzed using LC Model (Linear Combination of Model Spectra) software (version 6.3-1R; accessed on 1 December 2023) [99] with a basis set from the phantoms for TE = 68 ms.

**Figure 5:**
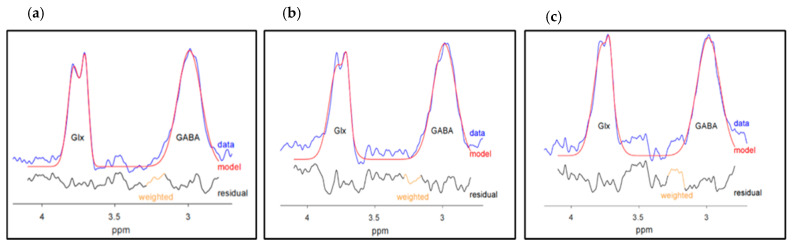
Modeling of GABA+ and Glx signals for (**a**) dmPFC/ACC, (**b**) dlPFC, and (**c**) insula. GABA-edited and Glx-edited spectra are shown in blue, the best-fit model is in red, and the black lines indicate the residuals between the blue and red lines. GABA+ = gamma-aminobutyric acid + macromolecules; Glx = glutamix; ppm = parts per million; dmPFC/ACC = dorsomedial prefrontal cortex/anterior cingulate cortex; dlPFC = dorsolateral prefrontal cortex.

**Figure 6:**
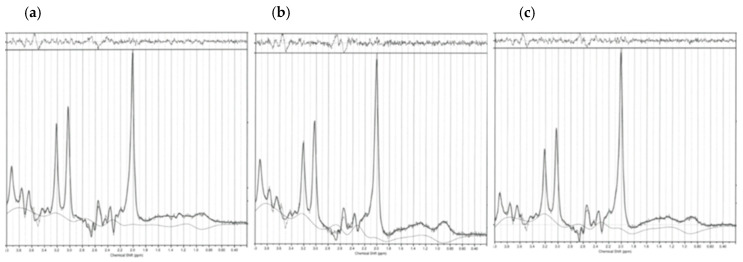
LC model editing-OFF spectra for (**a**) dmPFC/ACC, (**b**) dlPFC, and (**c**) insula. dmPFC/ACC = dorsomedial prefrontal cortex/anterior cingulate cortex; dlPFC = dorsolateral prefrontal cortex.

The data from the MEGA-PRESS editing-OFF was parsed, corrected for frequency, and combined using the FID-A toolkit [100] (accessed on 1 December 2023) before the LC Model analysis. Both water and metabolites were corrected for relaxation, the molal fractions of MR-visible water protons in CSF/GM/WM, macromolecule fractions, and for the approximate editing efficiency [101]. CSF/GM/WM voxel fractions resulted from the tissue segmentation obtained with the “fast” algorithm from FSL [102] (accessed on 1 December 2023). Figure 5 shows Gannet 3.1.5. GABA+ and Glx outputs, whereas Figure 6 shows the LC Model editing-OFF, MEGA-PRESS outputs for dmPFC/ACC, dlPFC, and the insula.

### 4.6. Statistical Analysis

Statistical analysis was performed using SPSS software, version 28.0 (IBM company, Armonk, NY, USA). The demographic variables and outcome measures were evaluated using descriptive statistics. The categorical variables were displayed as the number of observations and percentages. Alternatively, the continuous variables were shown by the number of observations, the means, and the standard deviations (SDs).

Poor quality of ^1^H MRS data was determined by inspecting their spectroscopic peaks for goodness-of-fit and comparing them to their quality parameters. The quality check for the MEGA-PRESS editing-OFF LC Model data included removing the data points with SNR > 10, FWHM < 0.1 ppm, and CRLB < 15%. The GABA+ and Glx data generated by Gannet were inspected for any outliers (based on standard deviations and the means), and these outliers were compared to their corresponding quality measures. The outliers with poor quality measures were removed as per Kurcyus et al., 2018, and Elsaid et al., 2023 [103,104].

Neurochemical concentrations between the groups were compared using the independent *t*-test and, where relevant, the non-parametric Mann–Whitney U test. The chi-square test was used to statistically compare the non-parametric variables. Linear regression analysis assessed the correlations between ^1^H-MRS metabolite concentrations and demographic and clinical outcome measures. To improve the accuracy of comparing Pearson’s correlation coefficients (*r*) between the groups, *r* values from both groups were converted to Fisher z scores [31]. Group differences in the Pearson correlational coefficients *r* were evaluated by converting the correlational coefficients determined for each study group using Fisher’s transformation (Equation (1)) and computing them using Fisher’s z test (Equation (2)). Equation (2) allowed computations of the Fisher z-score.

(1) $$r^{\prime }=\left(0.5\right)\log e\;\left|\frac{1+r}{1-r}\right|$$

(2) $$z=\frac{r1^{\prime }-r2^{\prime }}{\sqrt{\frac{1}{n1-3}+\frac{1\;}{n2-3}}}$$

In the equations above, *r* represents the sample correlational coefficient, and r′ is the transformed value of *r*. The sample size is indicated by *n*, whereas *z* refers to the z-score [31]. The adjustments for multiple comparisons were not performed because of the exploratory nature of our pilot study [105,106]. Thus, the results should be interpreted as indicators of potential trends and patterns to be confirmed in future studies. The significance of the data was set at *p* ≤ 0.05.

## 5. Conclusions

In this study, our main objectives were to measure GABA+ and Glx in dmPFC/ACC, dlPFC, and the insula in SAD. To our knowledge, our study was the first to report the imbalances of GABA+ in dlPFC, and Glx in the insula in individuals with SAD. The concentrations of other metabolites (NAA + NAAG, tCr, tCho, and mI) were also assessed in these three regions. Compared to the healthy controls, no differences in GABA+ or Glx levels were observed in dmPFC/ACC in SAD. Moreover, compared to the HCs, GABA+ levels were increased in dlPFC, whereas Glx was upregulated in the insula in SAD. Several explanations could be provided for these observations.

Increased GABA+ concentrations in dlPFC may be the byproduct of the aberrant GABA transporters (GAT); however, upregulated creatine-induced GABAergic activity could also explain these findings. In the insula, increased Glx with marginal decreases in GABA+ may have resulted from the dysfunction of Glu transporters or decreased GAD activity in the GABAergic inhibitory neurons. However, these proposed mechanisms need to be further investigated in SAD. Moreover, studies using higher magnetic fields (≥7T) should be conducted to determine the separate roles of Glu and Gln.

Our investigation of other metabolites suggests the presence of several pathological mechanisms in SAD. Disturbed mitochondrial function, cell membrane impairment, and energy production imbalances seem to be implicated in all three regions. The downregulated activity of GCPRs and their second messenger systems was associated with the pathological mechanisms in dmPFC/ACC and the insula. Gliosis in the astrocytes was suggested for dlPFC. However, these findings should be replicated in future studies.

## Figures and Tables

**Table 1:** Demographic and clinical variables.

	**SAD** **Group** (*n* = 26)	**Control** **Group** (*n* = 26)		
	***M (±SD)*** ***or %***	***M (±SD)*** ***or %***	***t or χ^2^***	***d or Phi***
***Demographic Variables***				
**Age (years)**	25.27 ± 5.01	25.15 ± 4.43	0.09	0.02
**Age of onset of SAD (years)**	12.67 ± 3.32			
**Sex (% female)**	61.5%	61.5%	0.00	0.00
**Comorbidities (%)**				
Clinical Features of AvPD	88.5%			
Lifetime MDD	26.9%			
Other (PDD, GAD)	19.2%			
**Education (% university of higher)**	57.7%	30.8%	3.45	−0.30
**Years of Education**	14.94 ± 2.71	15.90 ± 2.45	−1.34	−0.37
**Race (% Caucasian)**	42.3%	23.1%	1.40	0.21
**Race (% East Asian)**	30.8%	30.8%	0.00	0.00
**Employed or Student (%)**	92.0%	92.3%	0.00	0.00
**Last year’s income (% ≥$50,000**)	61.5%	42.3%	1.23	0.19
**Married/Common Law (%)**	30.8%	11.5%	1.85	−0.24
***Clinical Variables***				
**LSAS Total**	98.08 ± 13.56	7.77 ± 6.31	30.78 ‡	8.54
LSAS Anxiety	50.69 ± 7.95	5.23 ± 4.40	25.11 ‡	7.08
LSAS Avoidance	47.38 ± 8.22	2.54 ± 3.73	26.38 ‡	7.32
**SIAS**	49.62 ± 11.71	4.88 ± 4.54	18.17 ‡	5.04
**QIDS-16**	8.38 ± 5.12	2.23 ± 1.99	5.72 ‡	1.59
**SDS Total**	14.35 ± 7.47	0.85 ± 2.15	8.85 ‡	2.46
SDS Work/School	5.27 ± 3.01	0.35 ± 0.98	7.92 ‡	2.20
SDS Social Activities	6.31 ± 2.91	0.27 ± 0.88	10.14 ‡	2.81
SDS Family Life/Home Responsibilities	2.77 ± 2.71	0.27 ± 0.87	4.54 ‡	1.26
**WHOQoL BREF Physical**	50.92 ± 12.63	64.38 ± 7.66	−4.68 ‡	−1.29
**WHOQoL BREF Psychological**	54.38 ± 10.87	68.62 ± 8.49	−5.27 ‡	−1.46
**WHOQoL BREF Social**	61.58 ± 22.13	86.50 ± 10.90	−5.15 ‡	−1.43
**WHOQoL BREF Environmental**	71.81 ± 14.73	85.62 ± 11.02	−3.83 ‡	−1.06

‡ *p* ≤ 0.001; SAD = social anxiety disorder; M = mean; ± = plus/minus; SD = standard deviation; % = percentage; χ^2^ = chi-square test; t = independent *t*-test; d = Cohen’s d; Phi = Phi coefficient; AvPD = avoidant personality disorder; MDD = major depressive disorder; PDD = persistent depressive disorder; GAD = generalized anxiety disorder; LSAS = Liebowitz Social Anxiety Scale; SIAS = Social Interaction Anxiety Scale; QIDS-16 = Quick Inventory of Depressive Symptomatology; SDS = Sheehan Disability Scale; WHOQoL BREF = The World Health Organization Quality of Life scale Brief Version.

**Table 2:** Fisher z-scores indicating correlations between clinical variables and metabolite levels in dmPFC/ACC.

	GABA+ (i.u.)	Glx (i.u.)	GABA+/ Glx	NAA + NAAG (i.u.)	tCr (i.u.)	mI (i.u.)	tCho (i.u.)
**LSAS total**	−1.68	−0.84	−0.13	−1.80	−0.83	−1.69	−0.33
**LSAS Anxiety**	−2.40 *	−1.66	0.36	−2.04 *	−1.47	−1.79	−0.93
**LSAS Avoidance**	−0.66	0.17	−0.72	−1.18	0.13	−1.21	0.27
**SIAS**	−1.44	**−2.94 †**	1.73	−1.37	−0.81	−1.24	−1.15
**QIDS-16**	−0.03	0.14	0.07	−1.54	−1.30	−1.56	−1.46
**SDS total**	−1.49	−0.12	−0.90	−1.24	−2.43 *	−2.54 *	**−2.61 †**
**SDS Work/School**	−1.89	−0.53	−0.70	−1.70	**−2.86 ‡**	**−2.82 †**	**−3.11 †**
**SDS Social Life**	−1.44	−0.84	−0.60	−1.38	−2.03 *	−1.79	−1.58
**SDS Family Life**	−0.44	1.15	−1.57	−0.23	−0.95	−1.28	−0.92
**WHOQoL BREF, Physical**	−0.84	−0.39	0.00	0.86	0.75	1.73	1.82
**WHOQoL BREF, Psychological**	0.21	−0.43	−0.73	0.70	0.00	1.23	0.54
**WHOQoL BREF, Social**	1.03	−0.60	2.26 *	0.37	−0.69	0.14	−0.44
**WHOQoL BREF, Environmental**	−0.41	−0.37	0.40	0.59	0.67	1.16	1.47

* *p* ≤ 0.05; † *p* ≤ 0.01; ‡ *p* ≤ 0.001; i.u. = institutional units; LSAS = Liebowitz Social Anxiety Scale; SIAS = Social Interaction Anxiety Scale; QIDS-16 = Quick Inventory of Depressive Symptomatology; SDS = Sheehan Disability Scale; WHOQoL BREF = The World Health Organization Quality of Life scale Brief Version; dmPFC/ACC = dorsomedial prefrontal cortex/anterior cingulate cortex; GABA = gamma–amino butyric acid; Glx = glutamix; NAA = N-acetyl-aspartate; NAAG = N-acetyl-aspartyl-glutamate; tCr = total creatine; mI = myo-inositol; tCho = total choline. The number of social anxiety disorder (SAD) participants (*n*) examined for each metabolite was *n* = 25 for GABA+; *n* = 24 for Glx; *n* = 25 for NAA + NAAG; *n* = 26 for tCr; *n* = 26 for mI; *n* = 26 for tCho. The number of healthy controls (*n*) examined for each metabolite was *n* = 26 for GABA+; *n* = 24 for Glx; *n* = 25 for NAA + NAAG; *n* = 24 for tCr; *n* = 26 for mI; *n* = 24 for tCho.

**Table 3:** Fisher z-scores indicating correlations between clinical variables and metabolite levels in dlPFC.

	GABA+ (i.u.)	Glx (i.u.)	GABA+ /Glx	NAA + NAAG (i.u.)	tCr (i.u.)	mI (i.u.)	tCho (i.u.)
**LSAS total**	−0.98	1.32	−1.37	−1.06	−0.83	0.10	0.40
**LSAS Anxiety**	−1.66	0.65	−1.46	−1.23	−0.45	−0.13	0.50
**LSAS Avoidance**	0.27	1.74	−0.80	−0.45	−1.23	0.54	0.03
**SIAS**	−0.61	0.06	−1.29	−2.13 *	0.03	0.26	−0.24
**QIDS-16**	0.30	0.37	−0.31	−1.56	−0.40	1.00	−0.92
**SDS total**	1.33	0.59	0.92	−0.58	−0.60	0.45	−0.67
**SDS Work/School**	0.57	0.44	0.63	−1.46	−0.72	0.13	−1.00
**SDS Social Life**	1.60	0.92	0.62	−0.95	−0.35	1.03	−0.32
**SDS Family Life**	1.19	0.37	1.05	−0.20	−0.22	0.23	−0.07
**WHOQoL BREF, Physical**	−0.09	−0.75	1.09	1.36	0.20	−0.66	2.19 *
**WHOQoL BREF, Psychological**	−0.69	−0.91	0.70	1.28	−0.94	−2.15 *	0.25
**WHOQoL BREF, Social**	−1.88	−2.36 *	1.08	0.27	−2.15 *	−2.11 *	0.97
**WHOQoL BREF, Environmental**	−1.38	−1.46	0.60	0.62	−0.74	−0.63	1.07

* *p* ≤ 0.05; i.u. = institutional units; dlPFC = dorsolateral prefrontal cortex; LSAS = Liebowitz Social Anxiety Scale; SIAS = Social Interaction Anxiety Scale; QIDS-16 = Quick Inventory of Depressive Symptomatology; SDS = Sheehan Disability Scale; WHOQoL BREF = The World Health Organization Quality of Life scale Brief Version. GABA = gamma-aminobutyric acid; Glx = glutamix; NAA = N-acetyl-aspartate; NAAG = N-acetyl-aspartyl-glutamate; tCr = total creatine; mI = myo-inositol; tCho = total choline. The number of social anxiety disorder (SAD) participants (*n*) examined for each metabolite was *n* = 21 for GABA+; *n* = 22 for Glx; *n* = 25 for NAA + NAAG; *n* = 24 for tCr; *n* = 24 for mI; *n* = 24 for tCho. The number of healthy control participants (*n*) examined for each metabolite was *n* = 22 for GABA+; *n* = 22 for Glx; *n* = 22 for NAA + NAAG; *n* = 23 for tCr; *n* = 25 for mI; *n* = 24 for tCho.

**Table 4:** Fisher z-scores indicating correlations between clinical variables and metabolite levels in the insula.

	GABA+ (i.u.)	Glx (i.u.)	GABA+/ Glx	NAA + NAAG (i.u.)	tCr (i.u.)	mI (i.u.)	tCho (i.u.)
**LSAS total**	0.40	0.55	0.49	−0.79	−1.17	−1.23	−1.15
**LSAS Anxiety**	−0.23	−0.13	−0.33	−1.54	−2.23 *	−1.77	−1.57
**LSAS Avoidance**	−0.56	0.39	1.45	0.29	0.07	−0.43	−0.80
**SIAS**	−1.85	−1.01	−0.72	−0.92	−1.83	−1.25	−0.94
**QIDS-16**	1.21	−0.30	0.76	−0.20	−1.43	−1.78	0.28
**SDS total**	−0.07	−0.33	0.72	−0.20	−0.77	−0.97	−0.94
**SDS Work/School**	−0.37	−0.27	0.62	−0.96	−0.93	−0.87	−1.22
**SDS Social Life**	−0.30	−0.80	0.36	−1.07	−0.50	−0.41	−0.25
**SDS Family Life**	0.56	−0.95	1.23	0.13	−0.38	−0.95	−0.51
**WHOQoL BREF, Physical**	−1.61	−0.22	−0.89	0.23	0.85	1.50	0.94
**WHOQoL BREF, Psychological**	−0.29	0.27	0.21	0.76	0.55	0.77	−0.08
**WHOQoLBREF, Social**	−0.18	0.92	−0.66	0.62	−0.32	0.37	−0.24
**WHOQoL BREF, Environmental**	−0.69	0.24	0.18	1.74	1.69	1.96 *	0.52

* *p* ≤ 0.05; i.u. = institutional units; LSAS = Liebowitz Social Anxiety Scale; SIAS = Social Interaction Anxiety Scale; QIDS-16 = Quick Inventory of Depressive Symptomatology; SDS = Sheehan Disability Scale; WHOQoL BREF = The World Health Organization Quality of Life scale Brief Version. GABA = gamma-aminobutyric acid; Glx = glutamix; NAA = N-acetyl-aspartate; NAAG = N-acetyl-aspartyl-glutamate; tCr = total creatine; mI = myo-inositol; tCho = total choline. The number of social anxiety disorder (SAD) participants (*n*) examined for each metabolite was *n* = 23 for GABA+; *n* = 24 for Glx; *n* = 23 for NAA + NAAG; *n* = 24 for tCr; *n* = 24 for mI; *n* = 24 for tCho. The number of the healthy controls (*n*) examined for each metabolite was *n* = 26 for GABA+; *n* = 24 for Glx; *n* = 26 for NAA + NAAG; *n* = 26 for tCr; *n* = 26 for mI; *n* = 23 for tCho.

**Table 5:** MEGA-PRESS spectroscopic sequence parameters.

Scan Parameters	dmPFC/ACC	dlPFC	Insula
**Echo Time (TE)**	68 msec	68 msec	68 msec
**Repetition Time (TR)**	1500 msec	1500 msec	1500 msec
**Number of Acquisitions ^1^**	384	384	512
**Number of Excitations (NEX)**	8	8	8
**Number of Points**	4096	4096	4096
**Spectral Width**	5000 Hz	5000 Hz	5000 Hz
**Scan Time**	10.4 min	10.4 min	13.5 min

^1^ There were 192 editing-ON and 192 editing-OFF acquisitions for dmPFC/ACC and dlPFC, and 256 editing-ON and 256 editing-OFF acquisitions for the insula. A total of 16 unsuppressed water acquisitions were used for all three scans; dmPFC/ACC = dorsomedial prefrontal cortex/anterior cingulate cortex; dlPFC = dorsolateral prefrontal cortex; msec = milliseconds, min = minutes; Hz = Hertz; MEGA-PRESS = Mescher–Garwood Point Resolved Spectroscopy.

## Data Availability

The data are not publicly available due to their sensitive nature, and our ethical approval prevents us from sharing data beyond named collaborators. Further inquiries can be directed to the corresponding author: B.L.F.

## Supplementary Materials

The supporting information can be downloaded at https://www.mdpi.com/article/10.3390/ijms26146915/s1.
