# Supplementary material for: A Proton Magnetic Resonance Spectroscopy (1H MRS) Pilot Study Revealing Altered Glutamatergic and Gamma-Aminobutyric Acid (GABA)ergic Neurotransmission in Social Anxiety Disorder (SAD)
Source: Int J Mol Sci. 2025 Jul 18;26(14):6915. doi: 10.3390/ijms26146915 (PMC12295675; doi:10.3390/ijms26146915)
Supplement: Supplementary file 1 [file ijms-26-06915-s001.zip › Table S1 Supplemental_clear.pdf]

Supplemental Table S1. Fisher z scores indicating correlations between demographic variables and metabolite concentrations in dmPFC/ACC

	**Age**	**Sex[1]**	**Educ.[1]**	**Race (WC)[1]**	**Race (EA)[1]**	**Income[1]**	**Occ. Status[1]**	**Marital Status[1]**
**GABA+ (i.u.)**	-0.17	0.14	-0.10	0.10	0.38	**2.14***	0.66	0.72
**Glx (i.u.)**	-0.29	0.44	-0.10	0.28	0.37	1.54	0.20	-0.32
**NAA + NAAG (i.u.)**	-0.88	-0.26	0.07	1.48	-0.38	0.60	-0.28	0.49
**tCr (i.u.)**	-0.59	-0.78	-0.20	1.45	0.14	1.59	1.04	0.00
**mI (i.u.)**	-0.75	-1.56	-1.17	1.07	0.85	1.94	1.39	-0.45
**tCho (i.u.)**	-0.41	-1.06	-0.23	1.47	0.85	0.83	0.50	-0.96

*$p \leq 0.05$ [1]dummy coding for sex (0 = male, 1 = female; for the education level (0 = less than university, 1 = university education or higher); race (W), (0 = other, 1 = White); race (EA), (0 = other, 1 = East Asian); income (0 = <$50,000, 1 = ≥$50,000), occupational status (0 = unemployed, 1 = employer or student); marital status (0 = single or separated, 1 = married or common law). Educ. = education level; WC = White Caucasian; EA = East Asian; Occ. Status = occupational status; i.u. = institutional units; dmPFC/ACC = dorsomedial prefrontal cortex/anterior cingulate cortex; GABA = gamma–aminobutyric acid; Glx = (glutamate + glutamine); NAA = N-acetyl-aspartate; NAAG = N-acetyl-aspartyl-glutamate; tCr = total creatine; mI = myo-inositol; tCho = total choline. The number of SAD participants (*n*) examined for each metabolite was *n* = 25 for GABA+; *n* = 24 for Glx; *n* = 25 for NAA + NAAG; *n* = 26 for tCr; *n* = 26 for mI; *n* = 26 for tCho. The number of healthy controls (*n*) examined for each metabolite was *n* = 26 for GABA+; *n* = 24 for Glx; *n* = 25 for NAA + NAAG; *n* = 24 for tCr; *n* = 26 for mI; *n* = 24 for tCho.
