# Supplementary material for: A Proton Magnetic Resonance Spectroscopy (1H MRS) Pilot Study Revealing Altered Glutamatergic and Gamma-Aminobutyric Acid (GABA)ergic Neurotransmission in Social Anxiety Disorder (SAD)
Source: Int J Mol Sci. 2025 Jul 18;26(14):6915. doi: 10.3390/ijms26146915 (PMC12295675; doi:10.3390/ijms26146915)
Supplement: Supplementary file 1 [file ijms-26-06915-s001.zip › Table S2 Supplemental_clear.pdf]

Supplemental Table S2. Correlations between metabolite concentrations and SAD comorbidities in dmPFC/ACC

	**Clinical Symptoms of AvPD**	**Lifetime MDD**
**GABA+ (i.u.)**	-0.06	0.03
**Glx (i.u.)**	-0.27	0.03
**NAA + NAAG (i.u.)**	0.01	-0.10
**tCr (i.u.)**	-0.20	0.31
**mI (i.u.)**	-0.09	0.14
**tCho (i.u.)**	-0.02	0.15

[1] dummy coding for clinical symptoms of AvPD (0 = no, 1 = yes); lifetime MDD (0 = no, 1 = yes); AvPD = avoidant personality disorder; MDD = major depressive disorder; SAD = social anxiety disorder; i.u. = institutional units; dmPFC/ACC = dorsomedial prefrontal cortex/anterior cingulate cortex; GABA = gamma–aminobutyric acid; Glx = (glutamate + glutamine); NAA = N-acetyl-aspartate; NAAG = N-acetyl-aspartyl-glutamate; tCr = total creatine; mI = myo-inositol; tCho = total choline. The number of SAD participants (*n*) examined for each metabolite was *n* = 25 for GABA+; *n* = 24 for Glx; *n* = 25 for NAA + NAAG; *n* = 26 for tCr; *n* = 26 for mI; *n* = 26 for tCho. The number of healthy controls (*n*) examined for each metabolite was *n* = 26 for GABA+; *n* = 24 for Glx; *n* = 25 for NAA + NAAG; *n* = 24 for tCr; *n* = 26 for mI; *n* = 24 for tCho.
