# Supplementary material for: A Proton Magnetic Resonance Spectroscopy (1H MRS) Pilot Study Revealing Altered Glutamatergic and Gamma-Aminobutyric Acid (GABA)ergic Neurotransmission in Social Anxiety Disorder (SAD)
Source: Int J Mol Sci. 2025 Jul 18;26(14):6915. doi: 10.3390/ijms26146915 (PMC12295675; doi:10.3390/ijms26146915)
Supplement: Supplementary file 1 [file ijms-26-06915-s001.zip › Table S3 Supplemental_clear.pdf]

Supplemental Table S3. Fisher z scores indicating correlations between metabolite concentrations in dmPFC/ACC

	1	2	3	4	5
**1. GABA+ (i.u.)**					
**2. Glx (i.u.)**	1.59				
**3. NAA + NAAG (i.u.)**	1.63	1.49			
**4. tCr (i.u.)**	0.27	1.17	-0.21		
**5. mI (i.u.)**	0.40	**2.13***	-0.25	1.04	
**6. tCho (i.u.)**	0.17	0.11	-0.16	-0.43	1.04

*p≤0.05 i.u. = institutional units; dmPFC/ACC = dorsomedial prefrontal cortex/anterior cingulate cortex; SAD = social anxiety disorder; GABA = gamma–aminobutyric acid; Glx = (glutamate + glutamine); NAA = N-acetyl-aspartate; NAAG = N-acetyl-aspartyl-glutamate; tCr = total creatine; mI = myo-inositol; tCho = total choline. The number of SAD participants (*n*) examined for each metabolite was *n* = 25 for GABA+; *n* = 24 for Glx; *n* = 25 for NAA + NAAG; *n* = 26 for tCr; *n* = 26 for mI; *n* = 26 for tCho. The number of healthy controls (*n*) examined for each metabolite was *n* = 26 for GABA+; *n* = 24 for Glx; *n* = 25 for NAA + NAAG; *n* = 24 for tCr; *n* = 26 for mI; *n* = 24 for tCho.
