# Supplementary material for: A Proton Magnetic Resonance Spectroscopy (1H MRS) Pilot Study Revealing Altered Glutamatergic and Gamma-Aminobutyric Acid (GABA)ergic Neurotransmission in Social Anxiety Disorder (SAD)
Source: Int J Mol Sci. 2025 Jul 18;26(14):6915. doi: 10.3390/ijms26146915 (PMC12295675; doi:10.3390/ijms26146915)
Supplement: Supplementary file 1 [file ijms-26-06915-s001.zip › Table S4 Supplemental_clear.pdf]

Supplemental Table S4. Fisher z scores indicating correlations between demographic variables and metabolite concentrations in dlPFC

	Age	Sex[1]	Edu[1]	Race (WC)[1]	Race (EA)[1]	Income[1]	Occ. Status[1]	Marital Status[1]
**GABA+ (i.u.)**	1.15	-1.68	0.34	0.95	-0.77	**2.33†**	0.78	-1.65
**Glx (i.u.)**	-1.22	-0.76	-0.09	0.56	-0.92	0.75	0.22	-1.38
**NAA + NAAG (i.u.)**	0.77	-1.33	-0.55	1.40	-0.26	1.14	0.40	-1.09
**tCr (i.u.)**	1.75	-1.09	**-2.26***	-0.36	-0.42	0.99	1.01	1.26
**mI (i.u.)**	0.68	**-2.39***	-1.64	0.70	-0.44	0.37	1.66	-0.17
**tCho (i.u.)**	1.37	-0.85	-0.24	-0.98	1.71	1.15	1.90	-0.41

*p≤0.05; †p≤0.01; [1]dummy coding for sex (0 = male, 1 = female; for the education level (0 = less than university, 1 = university education or higher); race (W), (0 = other, 1 = White); race (EA), (0 = other, 1 = East Asian); income (0 = <$50,000, 1 = ≥$50,000), occupational status (0 = unemployed, 1 = employer or student); marital status (0 = single or separated, 1 = married or common law). Educ. = education level; WC = White Caucasian; EA = East Asian; Occ. Status = occupational status; SAD = social anxiety disorder; i.u. = institutional units; dlPFC = dorsolateral prefrontal cortex; GABA = gamma–aminobutyric acid; Glx = (glutamate + glutamine); NAA = N-acetyl-aspartate; NAAG = N-acetyl-aspartyl-glutamate; tCr = total creatine; mI = myo-inositol; tCho = total choline. The number of SAD participants (*n*) examined for each metabolite was *n* = 21 for GABA+; *n* = 22 for Glx; *n* = 25 for NAA + NAAG; *n* = 24 for tCr; *n* = 24 for mI; *n* = 24 for tCho. The number of healthy control participants (*n*) examined for each metabolite was *n* = 22 for GABA+; *n* = 22 for Glx; *n* = 22 for NAA + NAAG; *n* = 23 for tCr; *n* = 25 for mI; *n* = 24 for tCho.
