# Supplementary material for: A Proton Magnetic Resonance Spectroscopy (1H MRS) Pilot Study Revealing Altered Glutamatergic and Gamma-Aminobutyric Acid (GABA)ergic Neurotransmission in Social Anxiety Disorder (SAD)
Source: Int J Mol Sci. 2025 Jul 18;26(14):6915. doi: 10.3390/ijms26146915 (PMC12295675; doi:10.3390/ijms26146915)
Supplement: Supplementary file 1 [file ijms-26-06915-s001.zip › Table S5 Supplemental_clear.pdf]

Supplemental Table S5. Correlations between metabolite concentrations and SAD comorbidities in dlPFC

	**Clinical Symptoms of AvPD**	**Lifetime MDD**
**GABA+ (i.u.)**	**-0.54***	**0.48***
**Glx (i.u.)**	-0.09	0.18
**NAA + NAAG (i.u.)**	-0.28	0.27
**tCr (i.u.)**	-0.32	0.39
**mI (i.u.)**	**-0.41***	0.24
**tCho (i.u.)**	-0.20	0.30

*p≤0.05; [1] dummy coding for clinical symptoms of AvPD (0 = no, 1 = yes); lifetime MDD (0 = no, 1 = yes); AvPD = avoidant personality disorder; MDD = major depressive disorder; SAD = social anxiety disorder; i.u. = institutional units; dlPFC = dorsolateral prefrontal cortex; GABA = gamma–aminobutyric acid; Glx = (glutamate + glutamine); NAA = N-acetyl-aspartate; NAAG = N-acetyl-aspartyl-glutamate; tCr = total creatine; mI = myo-inositol; tCho = total choline. The number of SAD participants (*n*) examined for each metabolite was *n* = 21 for GABA+; *n* = 22 for Glx *n* = 25 for NAA + NAAG; *n* = 24 for tCr; *n* = 24 for mI; *n* = 24 for tCho. The number of healthy control participants (*n*) examined for each metabolite was *n* = 22 for GABA+; *n* = 22 for Glx; *n* = 22 for NAA + NAAG; *n* = 23 for tCr; *n* = 25 for mI; *n* = 24 for tCho.
