# Supplementary material for: A Proton Magnetic Resonance Spectroscopy (1H MRS) Pilot Study Revealing Altered Glutamatergic and Gamma-Aminobutyric Acid (GABA)ergic Neurotransmission in Social Anxiety Disorder (SAD)
Source: Int J Mol Sci. 2025 Jul 18;26(14):6915. doi: 10.3390/ijms26146915 (PMC12295675; doi:10.3390/ijms26146915)
Supplement: Supplementary file 1 [file ijms-26-06915-s001.zip › Table S6 Supplemental_clear.pdf]

Supplemental Table S6. Fisher z scores indicating correlations between metabolite concentrations in dlPFC

		1	2	3	4	5
**1.**	**GABA+ (i.u.)**					
**2.**	**Glx (i.u.)**	1.17				
**3.**	**NAA + NAAG (i.u.)**	1.64	**2.25***			
**4.**	**tCr (i.u.)**	**3.31‡**	1.00	**2.27†**		
**5.**	**mI (i.u.)**	**4.19‡**	0.31	1.67	1.61	
**6.**	**tCho (i.u.)**	0.47	0.29	1.63	**2.39***	**2.08***

*$p \leq 0.05$; †$p \leq 0.01$; ‡$p \leq 0.001$; i.u. = institutional units; dlPFC = dorsolateral prefrontal cortex; SAD = social anxiety disorder; GABA = gamma–aminobutyric acid; Glx = (glutamate + glutamine); NAA = N-acetyl-aspartate; NAAG = N-acetyl-aspartyl-glutamate; tCr = total creatine; mI = myo-inositol; tCho = total choline. The number of SAD participants (n) examined for each metabolite was n = 21 for GABA+; n = 22 for Glx; n = 25 for NAA + NAAG; n = 24 for tCr; n = 24 for mI; n = 24 for tCho. The number of healthy control participants (n) examined for each metabolite was n = 22 for GABA+; n = 22 for Glx; n = 22 for NAA + NAAG; n = 23 for tCr; n = 25 for mI; n = 24 for tCho.
