# Supplementary material for: A Proton Magnetic Resonance Spectroscopy (1H MRS) Pilot Study Revealing Altered Glutamatergic and Gamma-Aminobutyric Acid (GABA)ergic Neurotransmission in Social Anxiety Disorder (SAD)
Source: Int J Mol Sci. 2025 Jul 18;26(14):6915. doi: 10.3390/ijms26146915 (PMC12295675; doi:10.3390/ijms26146915)
Supplement: Supplementary file 1 [file ijms-26-06915-s001.zip › Table S7 Supplemental_clear.pdf]

**Supplemental Table S7**. Fisher z-scores indicating correlations between demographic variables and metabolite concentrations in the insula

| | Age | Sex[1] | Edu[1] | Race (WC)[1] | Race (EA)[1] | Income[1] | Occ. Status[1] | Marital Status[1] |
|---|---|---|---|---|---|---|---|---|
| **GABA+ (i.u.)** | -1.69 | 0.71 | **2.01*** | 0.49 | -0.59 | 0.43 | -1.10 | -0.26 |
| **Glx (i.u.)** | 0.26 | 0.70 | -1.12 | -1.63 | 0.88 | 0.10 | 0.10 | 0.43 |
| **NAA + NAAG (i.u.)** | 0.59 | -0.73 | 0.59 | 0.56 | 1.09 | -0.10 | -0.28 | 0.59 |
| **tCr (i.u.)** | 1.24 | -0.99 | 0.28 | 0.24 | 1.32 | 0.94 | 0.88 | -0.07 |
| **mI (i.u.)** | 0.69 | -0.98 | -1.27 | -0.03 | 1.59 | 0.04 | 0.84 | -0.27 |
| **tCho (i.u.)** | 0.31 | -1.29 | -1.10 | 0.60 | 1.00 | -0.87 | 1.14 | -1.54 |

*$p \leq 0.05$; [1]dummy coding for sex (0 = male, 1 = female; for the education level (0 = less than university, 1 = university education or higher); race (W), (0 = other, 1 = White); race (EA), (0 = other, 1 = East Asian); income (0 = <$50,000, 1 = ≥$50,000), occupational status (0 = unemployed, 1 = employer or student); marital status (0 = single or separated, 1 = married or common law). Educ. = education level; WC = White Caucasian; EA = East Asian; Occ. Status = occupational status; SAD = social anxiety disorder; i.u. = institutional units; n = number of participants; GABA = gamma–aminobutyric acid; Glx = (glutamate + glutamine); NAA = N-acetyl-aspartate; NAAG = N-acetyl-aspartyl-glutamate; tCr = total creatine; mI = myo-inositol; tCho = total choline. The number of SAD participants (*n*) examined for each metabolite was *n* = 23 for GABA+; *n* = 24 for Glx; *n* = 23 for NAA + NAAG; *n* = 24 for tCr; *n* = 24 for mI; *n* = 24 for tCho. The number of healthy controls (*n*) examined for each metabolite was *n* = 26 for GABA+; *n* = 24 for Glx; *n* = 26 for NAA + NAAG; *n* = 26 for tCr; *n* = 26 for mI; *n* = 23 for tCho.
