# Supplementary material for: A Proton Magnetic Resonance Spectroscopy (1H MRS) Pilot Study Revealing Altered Glutamatergic and Gamma-Aminobutyric Acid (GABA)ergic Neurotransmission in Social Anxiety Disorder (SAD)
Source: Int J Mol Sci. 2025 Jul 18;26(14):6915. doi: 10.3390/ijms26146915 (PMC12295675; doi:10.3390/ijms26146915)
Supplement: Supplementary file 1 [file ijms-26-06915-s001.zip › Table S8 Supplemental_clear.pdf]

Supplemental Table S8. Correlations between metabolite concentrations and SAD comorbidities in the insula

	Clinical Symptoms of AvPD	Lifetime MDD
**GABA+ (i.u.)**	0.19	0.12
**Glx (i.u.)**	0.19	-0.02
**NAA + NAAG (i.u.)**	0.09	-0.28
**tCr (i.u.)**	-0.14	0.09
**mI (i.u.)**	-0.11	-0.02
**tCho (i.u.)**	-0.29	0.02

[1] dummy coding for clinical symptoms of AvPD (0 = no, 1 = yes); lifetime MDD (0 = no, 1 = yes); AvPD = avoidant personality disorder; MDD = major depressive disorder; SAD = social anxiety disorder; i.u. = institutional units; GABA = gamma–aminobutyric acid; Glx = (glutamate + glutamine); NAA = N-acetyl-aspartate; NAAG = N-acetyl-aspartyl-glutamate; tCr = total creatine; mI = myo-inositol; tCho = total choline. The number of SAD participants (n) examined for each metabolite was n = 23 for GABA+; n = 24 for Glx; n = 23 for NAA + NAAG; n = 24 for tCr; n = 24 for mI; n = 24 for tCho. The number of healthy controls (n) examined for each metabolite was n = 26 for GABA+; n = 24 for Glx; n = 26 for NAA + NAAG; n = 26 for tCr; n = 26 for mI; n = 23 for tCho.
