# Supplementary material for: A Proton Magnetic Resonance Spectroscopy (1H MRS) Pilot Study Revealing Altered Glutamatergic and Gamma-Aminobutyric Acid (GABA)ergic Neurotransmission in Social Anxiety Disorder (SAD)
Source: Int J Mol Sci. 2025 Jul 18;26(14):6915. doi: 10.3390/ijms26146915 (PMC12295675; doi:10.3390/ijms26146915)
Supplement: Supplementary file 1 [file ijms-26-06915-s001.zip › Table S9 Supplemental_clear.pdf]

Supplemental Table S9. Fisher z-scores indicating correlations between metabolite concentrations in the insula

		1	2	3	4	5
**1.**	**GABA+ (i.u.)**					
**2.**	**Glx (i.u.)**	**2.49‡**				
**3.**	**NAA + NAAG (i.u.)**	0.44	0.36			
**4.**	**tCr (i.u.)**	0.43	-1.38	0.12		
**5.**	**mI (i.u.)**	0.39	-1.02	-0.15	-0.66	
**6.**	**tCho (i.u.)**	1.11	-0.35	0.00	0.36	0.36

‡$p \leq 0.001$; i.u. = institutional units; SAD = social anxiety disorder; GABA = gamma–aminobutyric acid; Glx = (glutamate + glutamine); NAA = N-acetyl-aspartate; NAAG = N-acetyl-aspartyl-glutamate; tCr = total creatine; mI = myo-inositol; tCho = total choline. The number of SAD participants (*n*) examined for each metabolite was *n* = 23 for GABA+; *n* = 24 for Glx; *n* = 23 for NAA + NAAG; *n* = 24 for tCr; *n* = 24 for mI; *n* = 24 for tCho. The number of healthy controls (*n*) examined for each metabolite was *n* = 26 for GABA+; *n* = 24 for Glx; *n* = 26 for NAA + NAAG; *n* = 26 for tCr; *n* = 26 for mI; *n* = 23 for tCho.
