# Supplementary material for: A Proton Magnetic Resonance Spectroscopy (1H MRS) Pilot Study Revealing Altered Glutamatergic and Gamma-Aminobutyric Acid (GABA)ergic Neurotransmission in Social Anxiety Disorder (SAD)
Source: Int J Mol Sci. 2025 Jul 18;26(14):6915. doi: 10.3390/ijms26146915 (PMC12295675; doi:10.3390/ijms26146915)
Supplement: Supplementary file 1 [file ijms-26-06915-s001.zip › Table S10 Supplemental_clear.pdf]

Supplemental Table S10. Comparison of GABA+ and Glx spectroscopic quality measures in dmPFC/ACC, dlPFC, and the insula

	SAD group		Control group			
**dmPFC/ACC**						
	M ±SD	*n*	*M ±SD*	*n*	*t*	*d*
**GABA+ SNR**	19.28 ± 4.32	25	19.86 ± 3.75	24	-0.50	-0.14
**GABA+ FWHM (Hz)**	21.22 ± 1.40	25	21.23 ± 1.27	24	-0.04	-0.01
**GABA+ fit error%**	4.19 ± 0.68	25	3.88 ± 0.72	24	1.57	0.45
**GABA+/H_20 fit error%**	4.25 ± 0.67	25	3.93 ± 0.70	24	1.59	0.46
**Glx SNR**	16.68 ± 4.39	23	16.91 ± 3.15	26	-0.22	-0.06
**Glx FWHM (Hz)**	18.62 ± 2.38	23	19.20 ± 2.67	26	-0.89	-0.23
**Glx fit error%**	4.85 ± 0.80	23	4.53 ± 0.87	26	1.33	0.38
**Glx/H_20 fit error%**	4.90 ± 0.78	23	4.58 ± 0.85	26	1.33	0.38
**Water FWHM (Hz)**	11.07 ± 1.33	25	11.41 ± 1.33	26	-0.92	-0.26
**dlPFC**						
	M ±SD	*n*	*M ±SD*	*n*	*t*	*d*
**GABA+ SNR**	19.20 ± 4.12	21	20.00 ± 3.29	22	0.00	0.00
**GABA+ FWHM (Hz)**	21.53 ± 1.15	21	20.62 ± 1.22	22	**2.51***	0.77
**GABA+ fit error%**	4.55 ± 0.75	21	3.95 ± 0.68	22	**2.75†**	0.84
**GABA+/H_20 fit error%**	4.60 ± 0.74	21	4.01 ± 0.67	22	**2.75 †**	0.84
**Glx SNR**	16.44 ± 3.87	22	15.34 ± 2.60	22	1.18	0.33
**Glx FWHM (Hz)**	18.81 ± 2.21	22	19.90 ± 1.86	22	-1.78	-0.53
**Glx fit error%**	5.65 ± 1.10	22	5.17 ± 0.97	22	1.53	0.46
**Glx/H_20 fit error%**	5.70 ± 1.10	22	5.22 ± 0.97	22	1.53	0.46
**Water FWHM (Hz)**	11.60 ± 1.29	22	11.72 ± 1.19	22	-0.32	-0.10
**Insula**						
	M ±SD	*n*	*M ±SD*	*n*	*t*	*d*
**GABA+ SNR**	14.87 ± 2.23	22	15.11 ± 2.30	26	-0.36	-0.10
**GABA+ FWHM (Hz)**	21.20 ± 1.23	22	20.70 ± 1.27	26	1.36	0.40
**GABA+ fit error%**	4.81 ± 0.70	22	4.98 ± 0.78	26	-0.78	-0.23
**GABA+/H_20 fit error%**	4.88 ± 0.71	22	5.05 ± 0.77	26	-0.80	-0.23
**Glx SNR**	13.58 ± 2.35	24	13.43 ± 2.03	24	0.25	0.07
**Glx FWHM (Hz)**	18.77 ± 2.00	24	18.08 ± 2.15	24	1.14	0.33
**Glx fit error%**	5.23 ± 0.83	24	5.64 ± 1.08	24	-1.49	-0.43
**Glx/H_20 fit error%**	5.29 ± 0.83	24	5.71 ± 1.07	24	-1.50	-0.43
**Water FWHM (Hz)**	11.11 ± 1.17	24	11.05 ± 1.07	26	0.18	0.05

*p ≤ 0.05; †p ≤ 0.01. GABA = gamma-amino butyric acid; Glx = glutamate + glutamine (glutamix); SAD = social anxiety disorder; dmPFC/ACC = dorsomedial prefrontal cortex/anterior cingulate cortex; dlPFC = dorsolateral prefrontal cortex; M = mean; SD = standard deviation; n = number of participants; t = independent t-test value; d = Cohen's d; SNR = signal to noise ratio; FWHM = full width at half maximum; Hz = Hertz; H_2O = water; % = percentage.
