# Supplementary material for: A Proton Magnetic Resonance Spectroscopy (1H MRS) Pilot Study Revealing Altered Glutamatergic and Gamma-Aminobutyric Acid (GABA)ergic Neurotransmission in Social Anxiety Disorder (SAD)
Source: Int J Mol Sci. 2025 Jul 18;26(14):6915. doi: 10.3390/ijms26146915 (PMC12295675; doi:10.3390/ijms26146915)
Supplement: Supplementary file 1 [file ijms-26-06915-s001.zip › Table S11 Supplemental_clear.pdf]

Supplemental Table S11. Comparison of MEGA-PRESS editing-off spectroscopic quality measures in dmPFC/ACC, dlPFC, and the insula

	SAD group		Control group			
**dmPFC/ACC**						
	M ±SD	*n*	*M ±SD*	*n*	*t*	*d*
**GM ratio**	0.62 ± 0.05	26	0.61 ± 0.04	26	-1.11	-0.03
**WM ratio**	0.16 ± 0.02	26	0.17 ± 0.03	26	-0.90	-0.25
**CSF ratio**	0.23 ± 0.059	26	0.22± 0.05	26	0.51	0.14
**GM / (GM + WM) ratio**	0.79 ± 0.02	26	0.08 ± 0.3	26	0.84	0.23
**NAA + NAAG CRLB%**	2.32 ± 0.56	25	2.28 ± 0.46	25	0.28	0.08
**tCr CRLB%**	1.96 ± 0.20	26	2.00 ± 0.00	24	-1.00	-0.27
**mI CRLB%**	5.54 ± 0.71	26	5.69 ± 0.74	26	-0.77	-0.21
**tCho CRLB%**	2,64 ± 0.49	25	2.79 ± 0.41	24	-1.17	-0.33
**H_2O SNR**	37.31 ± 4.34	26	37.04 ± 3.41	26	0.25	0.07
**H_2O FWHM (ppm)**	0.045 ± 0.008	26	0.042 ± 0.016	26	1.34	0.37
**H_2O Freq, drift (ppm)**	-0.045 ± 0.016	26	-0.042 ± 0.018	26	-0.65	-0.18
**dlPFC**						
	M ±SD	*n*	*M ±SD*	*n*	*t*	*d*
**GM ratio**	0.50 ± 0.05	26	0.49 ± 0.04	25	1.29	0.36
**WM ratio**	0.41 ± 0.07	26	0.44 ± 0.05	25	-1.62	-0.45
**CSF ratio**	0.09 ± 0.04	26	0.08 ± 0.03	25	1.29	0.36
**GM / (GM + WM) ratio**	0.55 ± 0.06	26	0.53 ± 0.05	25	1.57	0.44
**NAA + NAAG CRLB%**	2.04 ± 0.20	25	1.86 ± 0.47	22	1.64	0.50
**tCr CRLB%**	2.00 ± 0.00	24	2.04 ± 0.21	23	-1.00	-0.30
**mI CRLB%**	7.46 ± 1.35	24	7.08 ± 1.35	24	0.98	0.28
**tCho CRLB%**	2.92 ± 0.28	24	2.79 ± 0.51	24	1.05	0.30
**H_2O SNR**	39.73 ± 4.61	26	39.40 ± 4.06	25	0.27	0.08
**H_2O FWHM (ppm)**	0.0479 ± 0.010	26	0.0485 ± 0.012	25	-0.20	-0.06
**H_2O Freq. drift (ppm)**	-0.0367 ± 0.017	26	-0.0334 ± 0.021	25	-0.53	-0.15
**Insula**						
	M ±SD	*n*	*M ±SD*	*n*	*t*	*d*
**GM ratio**	0.61 ± 0.06	26	0.60 ± 0.05	26	0.47	0.13
**WM ratio**	0.19 ± 0.08	26	0.20 ± 0.03	26	-0.67	-0.19
**CSF ratio**	0.20 ± 0.05	26	0.20 ± 0.05	26	0.42	0.12
**GM / (GM + WM) ratio**	0.76 ± 0.08	26	0.75 ± 0.04	26	0.86	0.24
**NAA + NAAG CRLB%**	1.96 ± 0.37	23	2.08 ± 0.39	26	-1.11	-0.32
**tCr CRLB%**	2.04 ± 0.20	24	1.96 ± 0.20	26	1.42	0.40
**mI CRLB%**	7.79 ± 1.14	24	7.38 ± 0.94	26	1.38	0.39
**tCho CRLB%**	2.96 ± 0.36	24	2.87 ± 0.34	23	0.86	0.25
**H_2O SNR**	30.69 ± 3.43	26	31.96 ± 3.23	26	-1.34	-0.38
**H_2O FWHM (ppm)**	0.0458 ± 0.012	26	0.0414 ± 0.087	26	1.49	0.41
**H_2O Freq. drift (ppm)**	-0.0357 ± 0.032	26	-0.0378 ± 0.020	26	0.29	0.08

SAD = social anxiety disorder; MEGA-PRESS = Meshcher-Garwood Point Resolved Spectroscopy; dmPFC/ACC = dorsomedial prefrontal cortex/anterior cingulate cortex; dlPFC = dorsolateral prefrontal cortex; M = mean; SD = standard deviation; n = number of participants; t = independent t-test values; d = Cohen’s d; GM = grey matter; WM = white matter; CSF = cerebrospinal fluid; NAA + NAAG = N-acetyl aspartate + N-acetyl-aspartyl-glutamate; tCr = total creatine; mI = myo-inositol; tCho = total choline; CRLB = Cramer-Rao Lower Bounds; % = percentage; H_2O = water; Freq. = frequency; SNR = signal-to-noise ratio; FWHM = full width at half maximum; ppm = parts per million.
